# Potential determinants of health system efficiency: Evidence from Latin America and the Caribbean

**DOI:** 10.1371/journal.pone.0216620

**Published:** 2019-05-10

**Authors:** Rodrigo Moreno-Serra, Misael Anaya-Montes, Peter C. Smith

**Affiliations:** 1 Centre for Health Economics, University of York, York, United Kingdom; 2 Imperial College Business School, London, United Kingdom; University of Brescia, ITALY

## Abstract

This paper examines the levels of health system efficiency and their possible determinants across Latin American and Caribbean (LAC) countries using national-level data for those countries, as well as for other emerging and developed countries. The data are analyzed using data envelopment analyses and econometric advances that yield reliable estimations of the relationship between system efficiency and its potential determinants. We find that there is substantial room for efficiency improvements in the health system of most LAC countries. For example, LAC countries could improve life expectancy at birth by about five years on average at current public spending levels if they followed best practices. Furthermore, the paper assesses what factors amenable to policy act as the main possible levers for some countries to be able to translate a given level of health financing into better performance on access to care and health outcomes. Our econometric analyses suggest that efforts to increase health system efficiency could be focused in a few key policy areas associated with broader access to health services and better outcomes. These areas include general governance aspects, in addition to improvements in specific dimensions of the quality of health system institutions, notably stronger reliance on results-based management in the production of healthcare goods and services.

## Introduction

During most of the 2000s, the majority of Latin America and Caribbean (LAC) economies experienced sustained growth and improvements in social indicators, as shown by the evolution of real GDP growth and poverty reduction data [1]. Yet this trend has stalled in many LAC countries since the onset of the global financial crisis in 2008 and, despite recent progress, social inequalities remain pervasive in the region, with World Bank data on Gini indices placing 10 LAC countries among the 15 most unequal in terms of income distribution [1].

**Fig 1:**
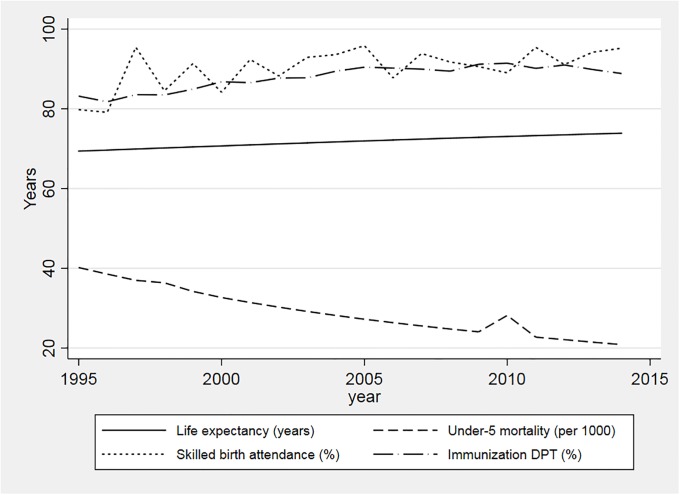
Selected health outcomes and coverage indicators in LAC (1995–2014).

The situation in the health sector closely mirrors the panorama described above. As a general rule, LAC countries have over a 20 year period achieved great improvements in health and well-being. Average life expectancy has been rising significantly for example, while under-five mortality rates have been consistently reduced (Fig 1). Health systems have likely been a crucial driver of this progress through a widening of access to necessary health services for citizens. Utilization measures commonly used as proxies for access to needed services, such as coverage of skilled birth attendance and immunization rates, have improved continuously during the 2000s (Fig 1).

Much of this health progress has taken place within the global push for universal health coverage (UHC). Yet progress towards UHC entails not only aggregate improvements in service access and health outcomes; at the center of this health agenda is also the drive to address unmet needs and health inequities [2]. A massive 125 million people in the LAC region still lack access to basic health services [3]. Health inequities persist among and within countries: some countries in the region exhibit far better progress than others judged by average improvements in key indicators, and in many instances health improvements have occurred favoring certain population groups at a disproportionate rate. For example, Haitians are expected to live 62 years on average, as compared to 79 years for Costa Ricans, and while under-five mortality is as low as 5.5 per 1,000 live births in Cuba, this figure reaches 39.4 per 1,000 live births in Guyana. Maternal mortality in rural areas in Bolivia is twice as high as in more urban areas, a pattern replicated in many other LAC countries [1, 3, 4].

The mixed health results achieved by LAC countries individually have taken place within a general context of increasing pressure on health budgets. Although the average share of public spending directed to the health sector among LAC countries has increased by as much as 41% between 2000–2012, cost pressures arising from technological advances and changes to demographic and epidemiological profiles mean that there will be continuing pressures for health expenditure to grow at rates above economic growth in many countries of the region [5]. In LAC, as in other regions of the world, countries vary significantly in terms of the population health indicators achieved for similar amounts of resources devoted to health (see Methods and data). Understanding the reasons for these differences in performance represents an essential step to the development of policies that ensure sustained progress towards UHC and health for all. This is an even more pressing need in the challenging economic context currently faced by many LAC countries, constraining public expenditures and public spending on health in particular.

There is now a good amount of evidence available about the relative levels of efficiency in the health sector of high-income countries [6, 7]. A recent review of the literature and other studies have concluded that there is evidence of widespread inefficiencies in the health sector of several OECD countries that help explain their differences in health attainment [8, 9]. These inefficiencies relate to factors including imbalances in physical and human resources relative to health needs, inadequate access to health prevention and promotion activities, and institutional factors such as deficient data collection and weak governance of the health system. By contrast, there is very little accumulated evidence on the degree and (particularly) determinants of health system efficiency in the LAC region as a whole, except for ad-hoc analyses of specific sectors in a few particular countries [10–15]. Some studies have attempted to measure health system efficiency using samples that include all world countries with available data, focusing often on comparisons of average efficiency across regions [16–21]. Invariably, these studies point to high levels of variation across countries in terms of efficiency performance and significant room for advances in health indicators through health system efficiency improvements.

However, such studies offer limited reliable evidence able to guide policy on how efficiency improvements could be achieved–either because of a lack of focus on identifying potential determinants of efficiency variations, or because of methodological limitations in attempting to do so. This applies not only to evidence regarding LAC countries but also to LMICs more generally. First, there is scant guidance in the cross-country literature about which factors under more direct policy control can be leveraged to improve health system efficiency. Even when previous studies have examined factors correlated with efficiency performance, such factors have usually been proxied by indicators that are too broad, or have very limited direct relationship with the functioning of the health system, to be of useful guidance for policymakers (e.g. urbanization, income distribution). Second, the influence of factors pertaining to the organization of healthcare delivery and the quality of health system institutions, found to be important for explaining variations in healthcare costs and efficiency in OECD countries [7, 22–24], has not been assessed systematically in a LMIC context using cross-country data. Third, although existing studies have examined health spending efficiency relative to some important health indicators (e.g. life expectancy), they have usually ignored other key outputs of the health system, particularly those related to access to care and equity of access to services–fundamental components of the UHC and SDG agendas [2, 25].

Our study uses LAC data to address some of the knowledge gaps mentioned above. Our goal is to identify the levels of health system efficiency and their possible determinants across LAC countries, as well as to provide insights into the quantitative influence of these potential determinants on observed levels of health system efficiency in the region. The measurement of ‘efficiency’ in the health sector has multiple connotations, depending on the entity under scrutiny and the decisions it is intended to support [26]. In this paper we address the concept of system level efficiency, understood as the extent to which resources devoted to the health sector succeed in securing health improvement, a prime concern of national policymakers charged with stewardship of the entire health system. We contribute to the knowledge base by: performing head-to-head comparisons of health system efficiency based on several outputs (health outcomes, care access and equity of access) for all LAC countries; benchmarking the results for LAC against other emerging and developed economies; and investigating possible system efficiency determinants related to the organization of healthcare financing and delivery, quality of governance, and quality of health system institutions in specific areas. This adds to our application of methodological advances that favor more reliable estimations of the relationship between system efficiency and its potential determinants.

## Methods and data

### Data envelopment analysis of health system efficiency

#### Methodology

Our study uses data envelopment analysis (DEA) to analyze efficiency in the achievement of health system objectives and its relationship with certain system characteristics at the country level. DEA is based on the economic principles of cost and production functions, and searches for the units that ‘envelop’ all other units on the basis of a composite estimate of efficiency [27]. For each unit–a country and its health system in our case–the ratio of actual to ‘optimal’ performance (or best practice) is referred to as inefficiency. As detailed below, we measure performance through health outcomes and access to care indicators, and the main input in our models is public health spending per capita. Throughout, our analyses use the ‘output orientation’ approach to DEA and the recommended model specification set out by Banker, Charnes and Cooper when variables are specified as ratios [28]. The output orientation indicates the extent to which a better performance on indicators of access to care and health outcomes could be obtained while still maintaining the same level of health expenditures. In simple terms, our approach identifies those countries that achieve the best performance on output indicators for their level of public health spending per capita, comparing other countries in relation to these ‘best performing’ countries.

Compared to statistical methods DEA has some attractive features, particularly in that it requires none of the stringent model testing that is required of statistical techniques. DEA studies need to be parsimonious in the selection of inputs however, as the inclusion of more inputs or constraints offers countries more potential ‘excuses’ for lower levels of performance, reducing the capacity to discriminate among countries and their health systems. Our study examines a range of modelling options in order to identify the sensitivity of judgments to different technical choices, including alternative sets of inputs and outputs.

#### Country samples

Our LAC sample contains annual information for the period 2006–2015 for 27 LAC countries. In order to be able to benchmark the efficiency performance of LAC countries, we use an extended sample that includes (in addition to the 27 LAC countries) 32 OECD countries and 12 non-OECD, non-LAC middle-income countries (MICs), totaling 71 countries with the inclusion of LAC. Although this extended sample forms the basis of our analysis, our presentation of results focuses on the performance of the LAC countries. Table A1 in S1 Appendix presents the full list of countries. The indicators used in our DEA estimations, along with their sources and five-year average values for the relevant periods, are described below and in Tables A2 and A3 in S1 Appendix.

#### Output indicators

We measure health system performance primarily with regard to broad indicators of population health status. These are complemented by key measures of progress towards UHC–service coverage indicators–agreed in the UHC monitoring framework [29], and we also acknowledge the relevance of assessing health system performance from the perspective of equity in access to necessary care [2]. Specifically, for the main estimations we run separate DEA models for each of the outputs below:

1. Health outcomes: life expectancy at birth (years); life expectancy at age 60 (years); under-five mortality rate (per 1,000 live births); and disability-adjusted life years lost (DALYs, all causes, age-standardized, per 100,000 population);
2. Access to services: skilled birth attendance (percentage of deliveries) and DPT immunization rate (percentage of children aged 12–23 months);
3. Equity of access to services: ratio poorest/richest wealth quintiles of births attended by skilled health staff; ratio rural/urban of births attended by skilled health staff.

The DEA methodology requires that output variables are measured in a way that indicates that ‘more is better’, so in the estimations we use the inverse of the under-five mortality rate and DALYs lost. We proxy general access to the health system through rates of utilization of services that should be provided to entire population groups, skilled birth attendance and DPT vaccination; together, these two proxies provide some indication of the conditions of access to the broader basket of services provided in a health system [30]. The most recent years for which the relevant country information is available varies by indicator. For output indicators we use five-year averages (2011–2015) in the DEA estimations, instead of their most recent values (except for DALYs and the equity of access measures, which are often available for just a single year between 2011–2015). The use of five-year averages is primarily for two reasons. First, the averaging procedure reduces the influence of extreme values (outliers) observed for countries due to, for example, one-off epidemiological or economic shocks and/or data measurement errors. Second, using averages allows us to explore data on specific indicators for more countries than using just the latest data (e.g. skilled birth attendance). We note that using the median output values instead of averages for the same period leads to very similar DEA efficiency scores, and virtually unchanged rankings of countries’ efficiency (the correlation coefficients between the two sets of efficiency scores for each output range from 0.89 to 0.99).

#### Input indicators

We assess the extent to which countries differ in the success with which their health system funds achieve a given level of performance. It is likely that the observed efficiency of LAC health systems is related principally to public funding, since governments typically have a major role in health system functions in the LAC region. Our study therefore uses public health expenditure per capita at PPP constant 2011 international dollars as the spending input in the main analyses. It consists of recurrent and capital spending from government (central and local) budgets, external borrowings and grants (including donations from international agencies and nongovernmental organizations), and social (or compulsory) health insurance funds. In robustness checks we use total health expenditure per capita or pooled prepaid health expenditure per capita as the main input instead.

**Fig 2:**
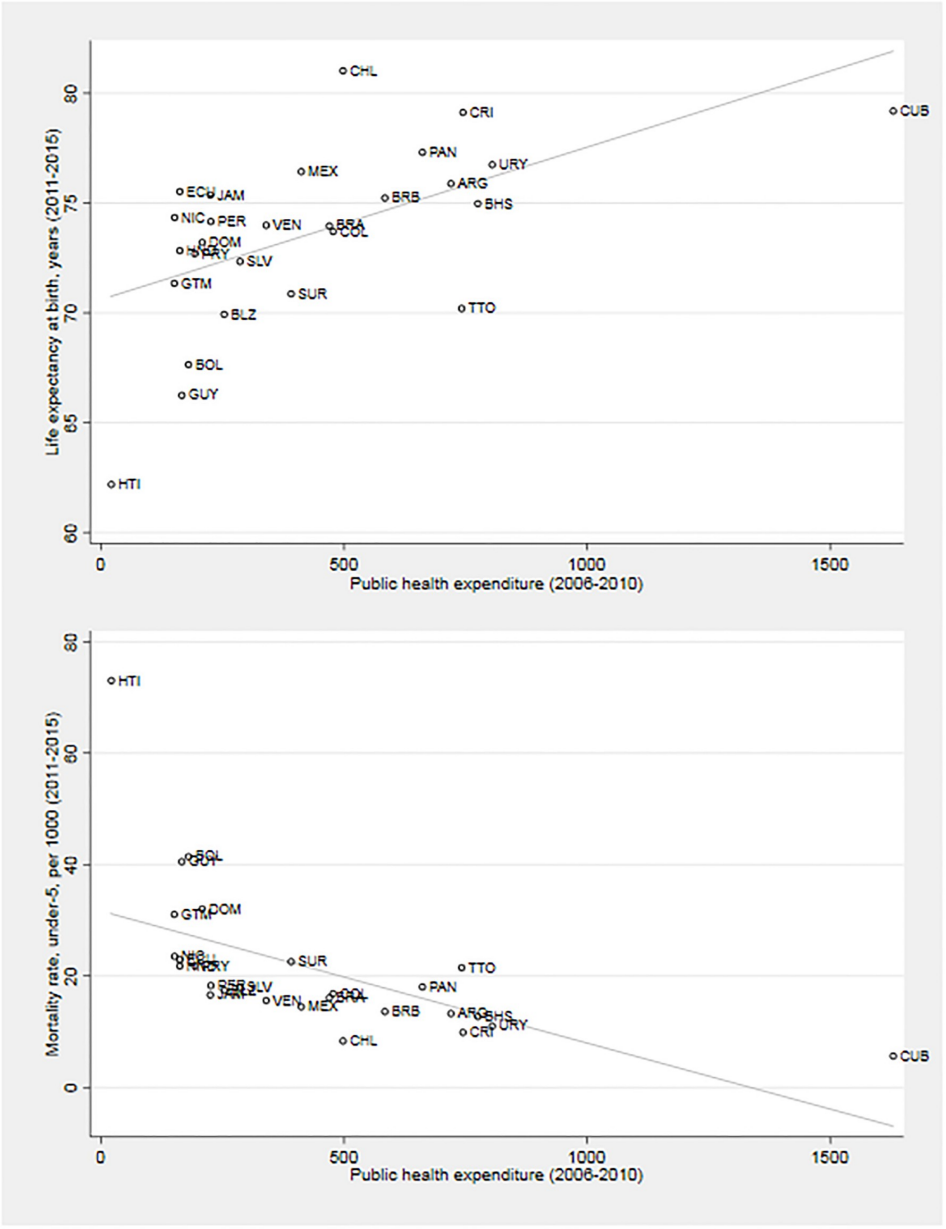
Life expectancy, under-five mortality and public health expenditure per capita in LAC.

We use five-year averages of our health expenditure measures in the DEA for the reasons discussed previously. Unlike outputs, however, we measure inputs as 2006–2010 averages. This is intended to reflect empirical evidence about time lags in the causal chain between health spending, changes in service access and quality, and consequent improvements in health outcomes at the country level [18, 31]. As can be seen in Fig 2, the health achievements of individual LAC countries vary considerably in the study period with regard to public health spending, with high spenders not necessarily being always the best performers. For example, Costa Rica and Trinidad and Tobago spend similar amounts per person but the former country records a much higher life expectancy and significantly lower under-five mortality than the latter.

#### External constraints (other inputs)

A country’s ability to maximize the impact of a given level of financial resources on health system outcomes is likely to be affected by economic and social development factors. Many of these factors may be uncontrollable influences on attainment and completely external to the health system. We therefore also include as additional inputs (constraints) in the DEA models:

1. National income: GDP per capita (constant 2011 PPP international dollars);
2. Age structure/demographics: population aged 65 and above (percentage of total).

As in the case of health spending measures, in the DEA estimations we use five-year averages 2006–2010 of GDP per capita and (the inverse of) the share of population aged 65. We also experimented with alternative specifications including further external constraints on performance, such as education achievement (average years of total schooling, age 15+), improved water source (percentage of population with access) and improved sanitation facilities (percentage of population with access). However, these indicators are highly correlated with GDP per capita in our sample (pairwise correlation coefficients ranging between 0.6 and 0.7, p<0.01), hence adding limited additional information to the models, and leading to an unwarranted inflation in the number of countries on the efficiency frontier. For these reasons, they have not been included in the analyses below. Finally, we would like to have explored data on lifestyle factors such as alcohol consumption and smoking prevalence as additional performance constraints, but these figures are unavailable for a large number of LAC countries.

#### The DEA models

We estimate three alternative DEA models for each health system output analyzed separately. Model (1) has public health spending per capita as the sole input. Model (2) includes public health spending per capita and GDP per capita. Finally, model (3) includes public health spending per capita, GDP per capita and population aged 65 and above as inputs.

### Regression analyses of potential health system efficiency determinants

#### Methodology

Because DEA efficiency scores are censored with an upper limit of one (or 100%), DEA studies have conventionally modeled the relationship between scores and potential determinants using a simple censored (Tobit) regression. This approach may lead to incorrect statistical inference if–as is often the case–there is high correlation among the estimated DEA efficiency scores [32]. In this case, using simple Tobit regressions could lead, for instance, to overstating the precision of estimates of efficiency determinants, leading to erroneous rejection of the null hypothesis of no statistical association between potential determinants and efficiency scores (which could be behind the high number of statistically significant associations identified in some studies; see e.g. [16]). A key issue is that the correlation pattern among DEA efficiency scores is typically complex and unknown.

We provide more reliable estimates of the relationships between various factors and health system efficiency by using an appropriate regression methodology to analyze DEA efficiency scores. The approach we adopt, developed by Simar and Wilson [32], accounts for the fact that DEA efficiency scores are bounded and corrects the standard errors obtained from conventional regression models such as Tobit, by simulating the unknown error correlation among efficiency scores and calculating bootstrapped standard errors. This method–henceforth Simar-Wilson–has been employed successfully in some previous efficiency studies focused on high-income countries [33] and, more recently, central European and central Asian countries [34]. Here we use Simar-Wilson cross-sectional regressions to estimate the degree of association between LAC countries’ average efficiency scores for each output as the dependent variable, and our potential efficiency determinants as explanatory variables. For these regressions, the estimating sample includes only LAC countries.

#### Potential efficiency determinants

In order to generate useful insights for policymaking, the potential efficiency determinants examined in the regressions analyses must refer to policy choices, as opposed to non-discretionary determinants of health system outputs (beyond health spending) that should have been captured in the initial DEA efficiency estimations [27]. Our goal is to explain statistically the DEA efficiency scores, investigating systematic associations between these scores and some discretionary characteristics of LAC health systems. There are significant limitations for LAC countries regarding comparative data about the organization of healthcare resources and system institutional factors. Despite these limitations, we have been able to gather data on some potentially important efficiency determinants, which can be grouped into three broad categories:

1. Organization of healthcare financing and delivery: out-of-pocket (OOP) health expenditure share (proportion of total health expenditure); hospital beds (per 1,000 people);
2. Quality of governance: governance indices for six dimensions (government effectiveness; voice and accountability; rule of law; regulatory quality; political stability and absence of violence/terrorism; control of corruption); we also construct an average governance index for the six individual dimensions. Higher indices indicate better performance.
3. Quality of health system institutions: indices for three dimensions (medium-term sectoral vision for the health system in line with the government plan; results-based management in the production of goods and services; sectoral information systems); we also construct an average health system institutional quality index for the three individual dimensions. Higher indices indicate better performance.

The precise definitions of the indicators above, their sources and sample averages are given in Tables A2 and A3 in S1 Appendix. The OOP share of health expenditure serves as an indicator of the reliance of health system financing on pooled prepaid revenue sources (or lack thereof). Revenue raising through prepaid sources such as general taxes and social insurance contributions has been shown to favor the production of better population health outcomes for a given health budget [35]. Therefore, a priori, we could expect to find a negative relationship between the OOP share indicator and system efficiency in our analyses. The hospital beds indicator, on the other hand, may provide information on the availability of physical resources for the provision of care in a health system; but it may also pick up other aspects such as a country’s reliance on hospital care compared to primary care. Thus the expected direction of relationship between hospital beds and efficiency is unclear a priori. Higher quality of governance in a country is expected to be positively related to the efficiency of its health system [36]. Lastly, the data on the quality of health system institutions includes assessments of e.g. the existence and alignment of health system planning with the overall government strategy, as well as availability of information systems in areas such as healthcare costs and quality. The three main indicators are computed from various sub-indicators (see Table A2 in S1 Appendix), arguably all of which can be expected to have a positive impact on efficiency in areas such as continuity of care, access to timely and clinically effective services, and spending on services [37].

We use the 2006–2010 country-specific average of the indicators 1–3 above in the estimations, to account for possible lags in the relationship between these indicators and efficiency levels, and to maintain consistency with the measurement of DEA inputs. The exceptions are the indicators of quality of health system institutions, for which we use the most reliable series only (year 2013).

## Results

### Health system efficiency levels within the LAC region

#### Main DEA results: Efficiency scores and rankings

For conciseness, the summary of the resulting DEA efficiency scores for the 24 models estimated (three DEA models for each of the eight health system outputs) is presented in Table A4 in S1 Appendix. The table shows the average efficiency scores across models (1), (2) and (3) for each country, by output indicator, along with the number of times each country ranks in the lowest or highest 25% of efficiency scores (i.e. worst and best performers, respectively) across all models.

The overall message from these estimations is that there is scope for efficiency improvements in the health system of many LAC countries, both in terms of access to care and population health indicators. Despite a few good LAC performers, most LAC countries (23) are located in the bottom half of the average efficiency ranking table across the whole sample. The best all-round performer in LAC is Chile, which is highly ranked (at 10^th^) for overall average efficiency. Chile’s high health system efficiency is driven by its solid performance with respect to generating good population health outcomes for its level of inputs. Other relatively good LAC performers in terms of overall health system efficiency include Barbados, Costa Rica and Cuba. By contrast, some LAC countries are consistently among the worst efficiency performers across the eight outputs examined, including Bolivia, Guatemala, Guyana and Panama. OECD countries occupy the large majority of the top 25% positions in terms of average efficiency score for the eight DEA outputs. However, given their typically higher input levels (especially health expenditures and national income), OECD countries act as efficient peers for LAC countries in only very few instances (e.g. Korea and Israel). Most peer pairings for LAC countries end up occurring among themselves and/or with some good performing MICs at different levels of inputs (e.g. China, Sri Lanka and Vietnam).

**Fig 3:**
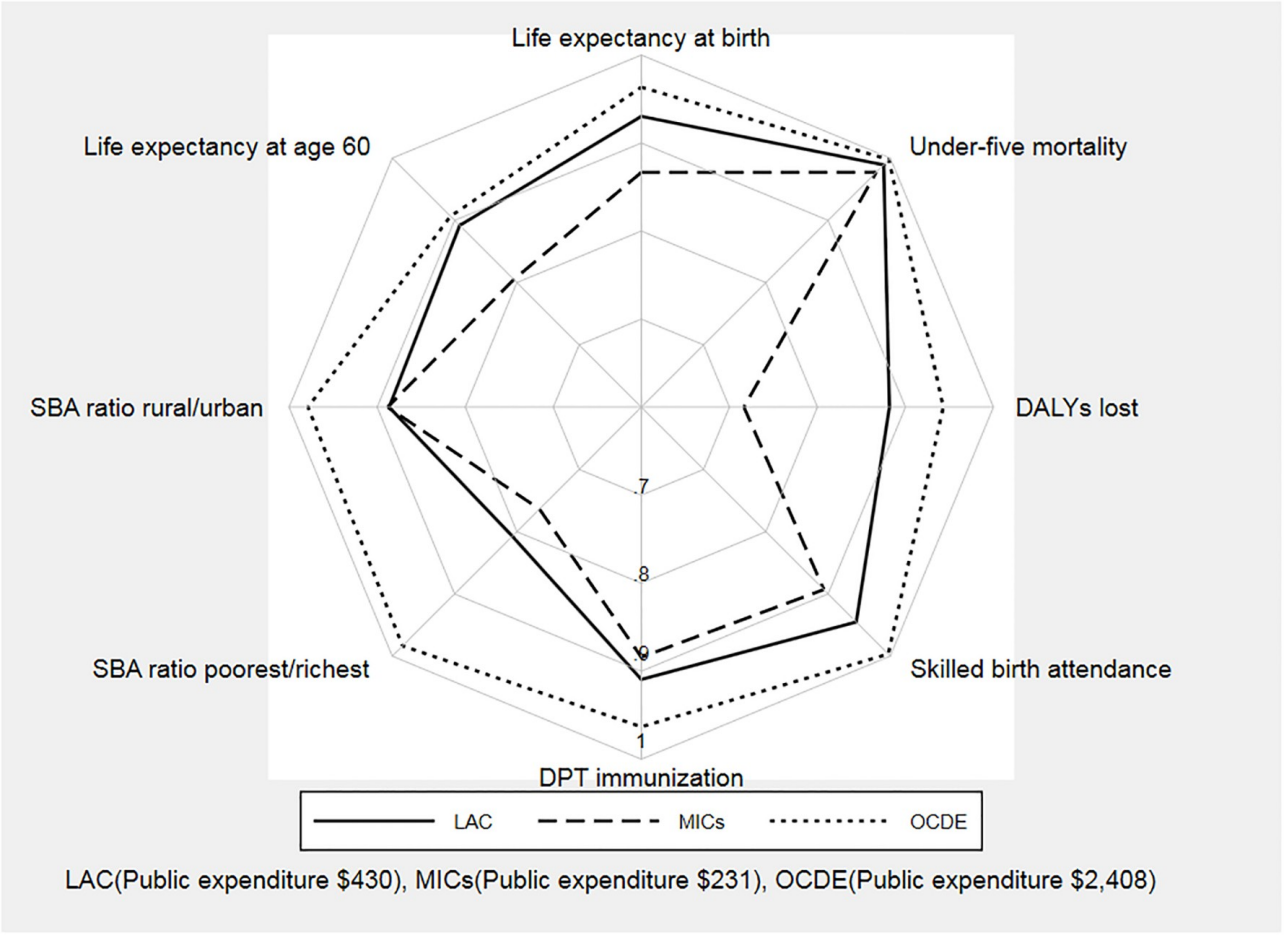
Comparison of average efficiency scores: LAC, OECD and MICs.

Fig 3 shows how the average efficiency scores per DEA output compare for the LAC, MICs and OECD groups. OECD health systems are the most efficient for all DEA outputs considered, while the non-LAC MIC group is the least efficient for all but one of the outputs. Important insights arise regarding the comparative efficiency of LAC health systems. On the positive side, the LAC region outperforms its comparable group of MICs for most health outputs, with an efficiency performance that is relatively close to that observed in the OECD for some health outcomes (life expectancy at age 60 and under-five mortality). On the negative side, LAC health systems perform particularly poorly as far as efficiency in providing equitable access to services is concerned.

The DEA results reveal the existing scope for many countries to make absolute improvements in health outcomes and access to care that can be very important from a health system perspective. We illustrate this by computing potential gains per country for each of our eight system output measures, where potential gains are calculated as the improvement a country could achieve on its average output indicator if the country moved to the corresponding estimated efficiency frontier (Table 1). In LAC, on average, life expectancy at birth could be increased by around 5 years–or 7% compared to the region’s average life expectancy–if countries moved from their current situation to the efficiency frontier, with corresponding figures of above 8 years for Bolivia, Guyana, Suriname and Trinidad and Tobago. Under-five mortality could be reduced on average by 10.5 deaths per 1,000 in LAC, which would correspond to reducing current average under-five mortality in the region by more than one-third, with potential reductions of between 22 to 25 deaths per 1,000 in Bolivia, Dominican Republic and Guyana. The indicators of access to the health system could also improve substantially if LAC countries moved to the efficiency frontier, with a potential increase of 4.5 percentage points on average for the skilled birth attendance rate, and a potential reduction of almost 12 percentage points in the gap between the skilled birth attendance rates of the poorest and richest citizens.

**Table 1:** Potential gains estimated by output indicator.

Country	Life expectancy at birth (years)	Life expectancy at age 60 (years)	Under-five mortality (per 1,000)	DALYs lost (per 100,000)	Skilled birth attendance (percentage points)	DPT immunization (percentage points)	Skilled birth attendance ratio poorest/richest (percentage points)	Skilled birth attendance ratio rural/urban (percentage points)
Argentina	5.6	3.01	8.0	6294	2.7	6.4	4.04	
Bahamas, The	6.7	3.44	8.6	10562	1.7	2.6		
Barbados	5.1	1.75	7.7	5083	1.3	7.5	2.56	1.17
Belize	7.2	2.34	8.4	7243	4.0	4.4	9.46	4.12
Bolivia	8.5	3.46	25.8	14060	11.2	4.3	20.00	18.37
Brazil	6.1	3.07	8.8	8676	2.0	3.5		5.26
Chile	2.0	1.05	1.8	1564	0.3	6.1		
Colombia	5.7	0.95	8.9	4696	0.9	9.1	13.24	11.35
Costa Rica	1.7	2.09	3.3	1945	1.7	8.1	3.78	3.69
Cuba	2.5	2.99	1.6	5775	0.4	1.1		1.56
Dominican Republic	4.4	1.29	22.8	5949	2.4	12.4	2.85	2.55
Ecuador	1.8	1.07	14.3	4990	6.7	13.5	16.37	12.97
El Salvador	5.5	1.73	8.5	10474	1.1	7.9	7.28	3.64
Guatemala	5.5	1.89	19.4	10556	22.8	14.5	23.27	21.97
Guyana	9.4	5.28	25.4	17895	4.6	3.9	12.91	5.31
Haiti	4.2	1.94	7.7	7160	2.0	5.0	2.91	3.18
Honduras	4.1	0.96	5.2	6400	11.3	11.8	16.63	14.28
Jamaica	2.5	2.08	7.6	8633	1.3	7.2	5.76	2.39
Mexico	4.4	2.74	7.2	5152	4.0	8.2		11.38
Nicaragua	2.5	1.76	6.4	5927	6.2	3.5	20.50	11.61
Panama	3.7	1.58	11.5	5935	7.1	15.1	20.80	18.10
Paraguay	4.5	2.04	10.2	5933	3.1	10.3		
Peru	3.8	0.53	9.4	3608	11.4	8.7	23.35	20.15
Suriname	8.3	1.38	15.0	7256		11.8	13.09	11.52
Trinidad and Tobago	10.4	5.10	16.8	15116	0.2	6.6	3.26	
Uruguay	4.5	2.64	6.3	7351	2.2	5.5		4.20
Venezuela, RB	5.4	1.09	7.7	6713	4.0	15.1		

As a side note, we also estimated multi-output DEA models where health system efficiency was assessed with respect to a set of three outputs included simultaneously, namely life expectancy at birth, DPT immunization and the ratio poorest/richest of skilled birth attendance (one multi-output model estimated for each of the input specifications in our base models 1–3). Unfortunately, the reduced ability to discriminate between more and less efficient countries in a multi-output DEA setting is as expected quite substantial in our data. Up to 44 out of the 59 countries with available data achieve maximum efficiency in the multi-output models. As DEA shows countries in the best possible light, good performance for just one output tends to drive several countries to the estimated efficiency frontier (for example, virtually all OECD countries are deemed fully efficient, chiefly due to their superior efficiency performance on service coverage and/or equitable access). Due to the dramatically reduced ability to discriminate between more and less efficient countries imposed by the mechanics of multi-output DEA as applied to our data, as well as the fact that in such a specification some countries secure 100% efficiency by attaching an unreasonably low weight on one or more outputs (hence contradicting widespread consensus that each of our output domains is an important element of health system performance [2, 29]), in this study we focus solely on the results of DEA and regression models estimated separately for each output of interest.

#### Potential determinants of health system efficiency

The above calculations of potential gains raise a crucial question from a policymaking viewpoint: what actions could ‘inefficient’ countries take to improve their health system outputs given their current levels of spending? This question requires some understanding of the main factors influencing measured levels of health system efficiency in LAC. We use Simar-Wilson cross-sectional regressions to estimate the degree of association between countries’ average DEA efficiency scores for each output (from the previous section) as the dependent variable, and our candidate efficiency determinants as explanatory variables. We focus below on the results for three main regression specifications (Tables 2 and 3). Results for a number of additional specifications are presented in Tables A5-A12 in S1 Appendix. The general conclusions are robust across all models so we concentrate on the main messages.

**Table 2:** Regression results of potential efficiency determinants: Health outcomes.

	Life expectancy			Life expectancy at age 60			Under-five mortality (per 1,000)			DALYs lost (per 100,000)		
	(1)	(2)	(3)	(1)	(2)	(3)	(1)	(2)	(3)	(1)	(2)	(3)
Out-of-pocket health expenditure (Perc.)	0.001		0.001	0.001		0.001	0.001		0.001	0.001		0.001
	(0.001)		(0.001)	(0.002)		(0.003)	(0.001)		(0.001)	(0.003)		(0.003)
Hospital beds (per 1,000 people)	0.002		−0.006	−0.009		−0.016	0.002		−0.001	0.016		−0.005
	(0.006)		(0.007)	(0.020)		(0.026)	(0.002)		(0.001)	(0.029)		(0.025)
Average governance quality		0.002	0.019		−0.015	0.016		0.005*	0.006*		0.039	0.061
		(0.013)	(0.015)		(0.476)	(0.068)		(0.003)	(0.003)		(0.050)	(0.060)
Average institutional quality		0.008	0.005		0.035	0.027		0.001	0.001		0.053	0.050
		(0.011)	(0.010)		(0.292)	(0.041)		(0.002)	(0.002)		(0.040)	(0.040)
Constant	0.912***	0.914***	0.904***	0.906***	0.856	0.880***	0.987***	0.989***	0.991***	0.856***	0.793***	0.759***
	(0.031)	(0.028)	(0.040)	(0.126)	(1.428)	(0.186)	(0.009)	(0.005)	(0.009)	(0.140)	(0.100)	(0.150)
Observations	27	24	24	27	24	24	27	24	24	27	24	24

Notes: Simar-Wilson models estimated with 1,000 bootstrap replications.

*p<0.1,

**p<0.05,

***p<0.01. Standard errors in parentheses. For under-five mortality, the very small coefficient and standard error for out-of-pocket health expenditure are rounded to three decimals.

**Table 3:** Regression results of potential efficiency determinants: Service access and equity of access.

	Skilled birth attendance			DPT immunization			Skilled birth attendance ratio poorest/richest			Skilled birth attendance ratio rural/urban		
	(1)	(2)	(3)	(1)	(2)	(3)	(1)	(2)	(3)	(1)	(2)	(3)
Out-of-pocket health expenditure (Perc.)	−0.017		−0.015	−0.002**		−0.001	−0.007		−0.005	−0.004		−0.005
	(0.018)		(0.013)	(0.001)		(0.001)	(0.018)		(0.007)	(0.006)		(0.004)
Hospital beds (per 1,000 people)	0.517*		0.227	0.005		−0.001	0.184		0.142	0.087		0.037
	(0.297)		(0.173)	(0.010)		(0.009)	(0.360)		(0.095)	(0.143)		(0.059)
Average governance quality		2.536*	0.052		0.030*	0.022		0.507	0.192		0.202	0.061
		(1.429)	(0.203)		(0.017)	(0.020)		(1.265)	(0.186)		(0.707)	(0.117)
Average institutional quality		0.262	0.135		0.001	0.002		0.008	0.051		0.002	0.019
		(0.590)	(0.110)		(0.013)	(0.013)		(0.244)	(0.097)		(0.143)	(0.050)
Constant	1.411	3.891	1.244	0.973***	0.910***	0.940***	0.892	1.129	0.817*	0.965**	1.024	1.035***
	(0.945)	(3.720)	(0.778)	(0.047)	(0.035)	(0.057)	(1.586)	(4.302)	(0.479)	(0.386)	(1.314)	(0.274)
Observations	26	23	23	27	24	24	19	18	18	21	19	19

Notes: Simar-Wilson models estimated with 1,000 bootstrap replications.

*p<0.1,

**p<0.05,

***p<0.01. Standard errors in parentheses.

#### Health outcomes

Overall, there are few systematic associations between efficiency scores for health outcomes (life expectancy measures, under-five mortality or DALYs lost) and any of the indicators of health system organization, governance and institutional quality. The one notable exception is the positive association between governance quality and system efficiency with respect to a key health outcome, under five-mortality (Table 2, columns 2–3). This result is robust to specification changes where the individual health system organization and institutional variables are added (Table A7 in S1 Appendix). We use the most conservative estimated coefficient in Table 2 (column 2) to provide some intuition on the magnitude of the association between governance and system efficiency with respect to under-five mortality. Our estimation implies that a one-unit increase in the average governance quality indicator is associated with an improvement of 0.005 in the efficiency score for under-five mortality. Consider a comparison between Panama, the ‘better’ country in the third quartile of governance quality (indicator = 0.100), and Uruguay, which scores in the top quartile of governance quality (indicator = 0.756). According to our DEA estimates in the previous sub-section, if Panama were to improve its governance quality to reach the position of Uruguay among the top 25% countries (approximately a one-standard deviation jump in the governance indicator), the resulting increase in Panama’s efficiency score would be equivalent to a reduction of 3.3 deaths in its under-five mortality rate, for the same level of public health spending.

#### Service access and equity of access

We find only weak associations between the service access efficiency scores and the OOP share or hospital beds indicators. While a couple of statistically significant results in Table 3 (column 1) suggest that higher shares of OOP expenditures (for DPT immunization) and lower number of beds (for skilled birth attendance) are associated with lower efficiency in the provision of skilled birth attendance and DPT immunization, these results are not robust to specification changes where governance and institutional indicators are included (column 3; see also Tables A9 and A10 in S1 Appendix). Similarly, there is only preliminary indication that better governance is associated with higher efficiency in providing access to necessary services for the general population. The coefficient on the average governance quality indicator is positive and statistically significant for skilled birth attendance and DPT immunization in column (2) but not in the full model (3). In spite of this lack of statistical significance in the full model, the link between governance and efficiency in service access is also found in further models where the health system institutional variables are examined individually instead of using an average index (Tables A9 and A10 in S1 Appendix, column 16). As a sense of the magnitudes involved, the results imply that if Panama were to improve its governance index to reach the level of Uruguay, the associated increase in Panama’s DPT immunization efficiency score would be equivalent to an increase of two percentage points in its DPT immunization rate, from the observed 81% to 83%, for the same level of public health spending.

For health system institutional quality, despite the lack of statistically significant results in the main models, an examination of the estimated coefficients for each of the institutional sub-indicators suggests a more nuanced picture, specifically for skilled birth attendance measures (see Tables A9 and A11 in S1 Appendix, columns 16–17). For skilled birth attendance provision and its ratio poorest/richest, there is indication that the extent of results-based management in the production of healthcare goods and services correlates with higher efficiency in providing both broader and more equitable service coverage. This positive result is counterbalanced in both cases by negative and statistically significant point estimates for the sectoral information systems variable, thus leading to point estimates for the average health system institutional quality variable that are positive yet relatively small and not statistically significant.

### Further robustness checks

In addition to the results for 17 alternative Simar-Wilson regression specifications for each output presented in S1 Appendix, in this section we further explore the robustness of our previous results to changing the key DEA input of interest for assessing efficiency, from public health expenditure to either (*i*) total health expenditure per capita or (*ii*) pooled prepaid health expenditure per capita. We compute revised DEA scores for cases (*i*) and (*ii*) separately and then run Simar-Wilson cross-sectional regressions as described previously. To save space, we present the full results of these analyses in S1 Appendix: Tables A13-A20 (total health expenditures) and Tables A21-A28 (pooled health expenditures).

#### Using total health expenditures as the main DEA input: Results

Moving to total health expenditure as DEA input again suggests that governance quality is linked to cross-country differences in service coverage efficiency: the results of the re-estimated Simar-Wilson regressions for the provision of skilled birth attendance and DPT immunization provide some additional indication in that respect (Tables A17 and A18 in S1 Appendix). The noteworthy change relative to our main regression results occurs for the skilled birth attendance ratio poorest/richest (Table A19): higher governance quality is now also linked to higher efficiency in providing more equitable access to skilled birth deliveries. On the other hand, the models with total health spending are once again unable to pick up any robust relationship between system efficiency and our system organizational variables, or average health system institutional quality.

#### Using pooled health expenditures as the main DEA input: Results

For this robustness check we change the main DEA input to pooled prepaid health expenditure per capita, defined as public (government) spending on health plus voluntary health insurance payments. This spending aggregate refers to funds paid by citizens before the need for medical care through sources such as taxation, social health insurance contributions and voluntary insurance plans. The causal influence of such pooled funds for improvements in access and population health has been established at the cross-country level [31, 35]. An argument for the use of pooled health spending in our analyses, instead of just public expenditures on health, is that in principle the consequent access and health improvements from better or higher spending on health can arise regardless of who makes the prepayment. Furthermore, within the wider context of push for UHC and progress around SDGs, pooled health financing indicates the prepaid resources that a nation is directly devoting to financial risk protection and effective access in the health sector, as opposed to out-of-pocket health payments made directly to providers at the point of service use. Therefore, measuring health system efficiency with respect to pooled spending frames the discussion more directly around how countries can target efficiency gains as a way to make better progress towards UHC.

The positive link between governance quality and health system efficiency is apparent once more when pooled health expenditure is used as the main input. In particular, the new regression results confirm a positive and statistically significant relationship between average governance quality and efficiency concerning under-five mortality achievement (Table A23 in S1 Appendix, columns 2–3). The estimated governance coefficients and standard errors have similar magnitudes to the corresponding estimates from the models using public health spending or total health spending per capita (the latter are only marginally statistically insignificant). The estimated governance effect is also positive and statistically significant for efficiency in DPT immunization coverage (Table A26 in S1 Appendix, column 2). Similarly to the case of total health expenditures, the regressions for pooled health expenditures suggest that higher governance quality is linked to higher efficiency in providing more equitable access to skilled birth attendance (Tables A27-A28 in S1 Appendix). The regressions for pooled health spending also lend some additional support to the suggestion that better health system institutions are associated with higher efficiency in service coverage judged by skilled birth attendance (Table A25 in S1 Appendix, column 3). As before, there is no robust relationship identified between efficiency and the out-of-pocket expenditure or beds per population proxies.

#### Estimations using a robust conditional approach: Results

Previous research has argued that, in spite of two-stage DEA approach being a commonly employed method to assess efficiency and its determinants, its ability to provide reliable inference depends on the validity of the key underlying assumption of ‘strong separability’, i.e. that the ‘environmental’ variables (in our case, the potential health system efficiency determinants) affect the distribution of inefficiencies but not the location of the production frontier [7, 38]. In order to explore whether our main empirical conclusions are changed should the ‘strong separability’ assumption fail in our context, we re-estimate our models using conditional order-m efficiency estimators, whereby the estimation of the efficiency frontier and of potential efficiency determinants is undertaken simultaneously, non-parametrically and with better convergence properties than with other DEA estimators; for details see [7] (we thank an anonymous referee for this suggestion). We perform a separate conditional order-m efficiency estimation for each of our eight output variables. The variables included as inputs in each model are, in addition to public health spending per capita, GDP per capita and population aged 65 and above. As environmental variables we include the out-of-pocket health spending share, hospital beds, the average governance index and the average health system institutional quality index. The results of the conditional order-m estimations are shown in Table A29 and Figs A1-A8 in S1 Appendix.

Overall, the new estimations actually strengthen our conclusion that both better governance and higher quality of health institutions are associated with higher health system efficiency. For the eight outputs, we find that better average governance is linked to better efficiency performance, in most cases at the 1% level of statistical significance. We also find that higher quality of health institutions is unequivocally and statistically significantly linked to better efficiency scores with respect to key indicators of health (life expectancy at birth and under-five mortality), access to care (DPT immunization) and equity of access (the rural/urban ratio of skilled birth attendance), at the 5% statistical significance level or lower.

The only noteworthy changes to our main conclusions refer to the relationships of efficiency with our proxies for the organization of healthcare financing and delivery. Firstly, the conditional order-m models find that health systems that rely less on out-of-pocket spending (as share of total health expenditure) tend to produce better health outcomes for a given level of health financing, judged by all our indicators of life expectancy, mortality and morbidity. This lends some support to previous findings that pooled prepaid financing is normally more efficient in producing better population health than OOP health financing [35]. Secondly, the new estimates suggest that higher numbers of hospital beds per person are associated with higher system efficiency in generating better health (except mortality), service access (DPT immunization) and equity of access (ratio poorest/richest of skilled birth attendance), which could be interpreted as a general indication that higher health system efficiency is linked to wider availability of health infrastructure for the population.

Taken together, the results of the conditional order-m estimations strongly reinforce the conclusions of the main analyses regarding the positive relationship between health institutional quality and general governance on the one hand, and health system efficiency on the other. If anything, our main (two-stage) estimation approach is in fact providing lower bounds of efficiency effects, particularly for the potential influence of health institutional variables. In the Discussion section below, we therefore focus mostly on the results of our main two-stage analyses for all potential efficiency determinants so as to remain on the more “conservative” side.

## Discussion and conclusions

The first message from our analyses is that there appears to be substantial room for efficiency improvements in the health system of most LAC countries. For example, LAC countries could improve life expectancy at birth by about 5 years on average at current spending levels if they followed best practices. This magnitude is larger than the comparable estimate of about 3 years for LAC countries found by Grigoli and Kapsoli [18] (it is in fact similar to their estimate of potential average gain found for African economies) and much larger that an estimate of 1.8 potential extra years on average for OECD countries [8]. Furthermore, despite relatively good efficiency performance for under-five mortality and life expectancy at age 60, LAC health systems perform especially poorly compared to OECD ones and other MICs regarding the provision of equitable access to services for their levels of health spending.

These potential efficiency gains should be sought not only as a matter of public sector accountability (since health financing in most LAC countries relies heavily on public sources), but also because the resulting health gains could represent a crucial step in the progress of less efficient countries towards UHC. However, advocating reductions in health spending in LAC to achieve these efficiency gains is not a policy implication arising from our analyses. The fact that even the highest health spenders in LAC are not necessarily close to a ‘health production frontier’ (e.g. defined by the health attainment of OECD countries) means that reducing current health expenditure levels–a key input to enhance the quantity and quality of health services–would likely be counterproductive for health development in the region [31, 35]. Instead, the policy implication for health system efficiency is that LAC countries should seek ways to improve the health outcomes and coverage indicators achieved for their *current* levels of resources, rather than seek to reduce their health expenditure levels, which are already low compared, for instance, to OECD countries. Improving population health and equitable access to services by spending currently available resources more wisely represents a ‘low-hanging fruit’ for health financing in LAC, which can be used to relieve acute budgetary constraints, as well as to make a stronger case for increases in the share of government resources devoted to the health sector.

A second message from our study is that efforts to increase health system efficiency could be focused in a few key policy areas linked empirically to cross-country efficiency variations in LAC, including general governance aspects. The relationship between better governance and higher system efficiency is apparent in several of our estimations, both in the main analyses and robustness checks. A closer look at the most efficient OECD and MIC peers reveals that these countries score highly in aspects such as government effectiveness; transparency and citizens’ participation in policymaking; and regulatory quality. All these factors are likely to favorably influence the functioning and efficiency of the public sector [36], which plays a pivotal role for the organization and functioning of most health systems in LAC. Some of the more health efficient LAC countries have indeed made important advances in improving public sector regulation, transparency and accountability to citizens–for instance, by embarking in sensible open government reforms–and these may make policymaking and public spending processes more efficient. In Costa Rica, the implementation of a General Comptroller’s Office web-based tool allowing citizens to monitor public spending is believed to have favored better public spending targeting, through fostering citizens’ engagement and social control of government expenditures [39]. Similar initiatives have been adopted in Chile and Uruguay, and may be behind these countries’ relatively high public sector efficiency performance–and health sector efficiency–within the region [40]. The experiences of Chile, Costa Rica and Uruguay of developing an e-government system and advancing on e-procurement processes could offer leads to other LAC countries about promising initiatives for improving governance and public spending efficiency in healthcare.

A third takeaway message from our empirical results is that greater health system efficiency in LAC could also be stimulated by improvements in specific dimensions of the quality of health system institutions. The general importance of health institutions for system efficiency is suggested somewhat more conservatively in the main estimations, yet it is reinforced, and clearly strengthened, in our more disaggregated analyses and robustness tests. The most compelling evidence about the importance of particular health institutions refers to the positive association of efficiency in the provision of broader and more equitable access to health services with stronger reliance on results-based management in the production of healthcare goods and services. The results-based management indicator examined here is a composite measure encompassing performance within different sub-areas of the public healthcare system. A key set of these sub-areas is the planning and active monitoring of annual/multi-annual targets for the provision of healthcare goods and services, which seems to highlight the potential benefits of initiatives to incorporate a medium-term perspective in the general and health budgets. Many OECD countries including Korea–which is often identified in our DEA estimations as an efficient peer for ‘high-spender’ LAC countries–have implemented sound medium term expenditure frameworks (MTEFs) to help manage expenditures across government sectors and ensure fiscal discipline, as well as to give ministries time to adjust and better plan operations. The Korean five-year MTEFs, for example, are perceived to have improved fiscal responsibility, spending planning capacity, and spending efficiency across all areas of government [41, 42]. In LAC, Costa Rica has also made improvements to planning processes particularly around benefit package expansions and human resource management, in a bid to boost both the efficiency and equity of health spending by the single insurer [39]. This may already have been reflected at least in part in the country’s relatively high system efficiency scores for the provision of general and equitable health service coverage. Experience shows, however, that how a medium-term planning process is actually adhered to (and used to inform decisions) is far more relevant for improving efficiency performance than whether there is any medium-term planning in place. General characteristics shared by successful experiences include clear and transparent criteria to identify spending priorities, and implementation of annual reviews of sector-level progress [43].

Another sub-area included in our results-based management indicator is the presence of personnel remuneration and evaluation systems that incentivize results. In this regard, our results concur with findings for OECD countries linking well-designed performance-based provider payment systems with efficiency gains [37]. Chile and Uruguay, two of the most efficient LAC health systems, have both adopted some degree of pay-for-performance, notably for the reimbursement of primary care personnel in the public sector (e.g. risk-adjusted capitation complemented by performance-based payment in Uruguay). This is a particularly relevant area for policy action in the LAC region, where several health systems rely primarily on reimbursement methods that do little to stimulate efficient healthcare spending, such as fee-for-service schemes operating within soft budgets [44].

By using LAC data to assess the issues above, this study goes one step further than much of the available literature towards assessing what (policy-amenable) factors act as the main possible determinants–and to what extent–for some countries to be able to translate a given level of health financing into better performance on access and health outcomes, than that achieved by other countries. We use robust regression methodologies and explicitly examine indicators of overall and equitable access to services, which is crucial for health policy in LMICs (and specifically in LAC), unlike previous studies that have tended to focus exclusively on population averages of mortality or life expectancy measures. Nevertheless, as with any empirical work, our study has limitations imposed by unavoidable methodological choices and data constraints. Firstly, in examining empirically some potential determinants of cross-country differences in health system efficiency, the key word is ‘potential’. Although it seems sensible in theory to expect factors like governance and health institutions to influence system efficiency directly, with our data we cannot ascertain whether the estimated relationships do indeed represent *causal* links. We can only claim that our Simar-Wilson estimates reflect associations in the data, suggesting that the efficiency performance of LAC countries and some of its potential determinants move together systematically. Country-case studies based on ‘natural experiments’ and finer data would be better positioned to identify causal relationships. Notwithstanding this caveat, it is reassuring that our estimates of links between health system efficiency and key potential determinants generally follow the direction one would expect for these relationships, based on theory and available evidence for other contexts.

Secondly, analytical constraints imposed by data availability for LAC countries have been alluded to in previous sections and are a general issue among LMICs. Stepped up efforts by international organizations are needed to compile comprehensive and harmonized data over time on health system spending, throughput and outcome indicators, disaggregated among others by levels of care provision and population groups, to allow more comprehensive analyses of health system performance. Data disaggregated at these levels are typically unavailable as comparable time series for LMICs. A final and crucial data issue for our analyses has been limited information about the institutional characteristics of LAC health systems. Despite being helpful (and a step ahead of what is available for most LMICs), the PET (PRODEV) health institutional dataset that we examine for LAC was designed with the aim of evaluating countries’ capacity to adopt results-based public management, and not specifically to offer a comprehensive assessment of health system organization and functions across LAC countries. The sectoral information systems indicator, for instance, refers primarily to the mere existence of any such system in a given country, which may help explain some counterintuitive empirical results for that variable.

Surveys that collect harmonized information on health system characteristics across several countries have represented useful tools for investigations of the main drivers of health expenditures and system efficiency in other settings. A notable example is the Health Systems Characteristics Survey in the case of OECD countries [5, 22]. This collects information on aspects including the organization of healthcare financing and delivery, provider payment schemes, user choice, regulation of healthcare supply and prices, and use of health technology assessment, among many others. Similar surveys for the LAC region would enable future comparisons of health system efficiency to offer more granular evidence to better inform policy. For example, some ‘efficient’ LAC countries (e.g. Chile, Uruguay) have moved away from historic budgets and/or uncapped fee-for-service schemes to pay hospitals, which tend to encourage inefficient resource use (cf. [37] for a review), towards case-based reimbursement alongside pay-for-performance elements. Similarly, although LAC health systems in general are still heavily reliant on specialist, hospital-based and more expensive curative care, relatively efficient LAC health systems including Costa Rica and Uruguay have long offered comprehensive primary care coverage to citizens, while Chile introduced reforms in 2005 to reinforce cost-effective primary care as the center of healthcare networks [44]. Unfortunately, without systematically collected and comparable time series data for most LAC countries on aspects like health spending and provision by levels of care, or provider reimbursement arrangements, we are unable to move from preliminary speculation about the link between said institutional factors and system efficiency, toward finer policy guidance on these particular issues based on empirical evidence. We believe, however, that the empirical analyses that we are able to undertake in this study do offer policy-relevant insights for countries seeking to increase efficiency in the use of existing health resources and reduce cost pressures in the health system, while still making progress towards UHC/SDG goals and better population health.

## Supporting information

S1 Appendix (DOCX)

## References

[1] World Bank. World Development Indicators [cited 2018 Feb 26]. http://databank.worldbank.org/data/reports.aspx?source=World-Development-Indicators.

[2] World Health Organization. Health systems financing: the path to universal coverage. Geneva: WHO; 2010. 10.2471/BLT.10.078741 PMC2878164 20539847

[3] Pan American Health Organization. EquiLAC II Project [cited 2017 Nov 21]. http://www.paho.org/hq/index.php?option=com_content&view=article&id=2675&Itemid=2077&lang=en.

[4] Etienne CF. Editorial: Equity in health systems. SciELO Public Health. 2013;33(2):79–80. 10.1590/s1020-49892013000200001 23525336

[5] de la Maisonneuve C, Oliveira Martins J. Public spending on health and long-term care: a new set of projections OECD Economic Policy Papers 6. Paris: OECD; 2013.

[6] Cylus J, Papanicolas I, Smith PC. Health system efficiency: how to make measurement matter for policy and management. London: European Observatory on Health Systems and Policies; 2016. 28783269

[7] Gearhart R. The impact of secondary environmental variables on OECD healthcare efficiency: a robust conditional approach. The BE Journal of Economic Analysis & Policy. 2019 10.1515/bejeap-2018-0063

[8] Medeiros J, Schwierz C. Efficiency estimates of health care systems Economic Papers 549. Brussels: European Commission; 2015.

[9] Gearhart R. The robustness of cross-country healthcare rankings among homogeneous OECD countries. 2016;19(1):113–43.

[10] Hernandez AR, Sebastian MS. Assessing the technical efficiency of health posts in rural Guatemala: a data envelopment analysis. Global Health Action. 2014;7(1):23190. 24461356 10.3402/gha.v7.23190 PMC3901389

[11] Ligarda J, Ñaccha M. La eficiencia de las organizaciones de salud a través del análisis envolvente de datos: microrredes de la Dirección de Salud IV Lima Este 2003. UNMSM. Facultad de Medicina, 2006.

[12] Ramírez-Valdivia MT, Maturana S, Salvo-Garrido S. A multiple stage approach for performance improvement of primary healthcare practice. Journal of Medical Systems. 2011;35(5):1015–28. 10.1007/s10916-010-9438-7 20703756

[13] Ruiz-Rodriguez M, Rodriguez-Villamizar LA, Heredia-Pi I. Technical efficiency of women’s health prevention programs in Bucaramanga, Colombia: a four-stage analysis. BMC Health Services Research. 2016;16(1):576 10.1186/s12913-016-1837-0 27737662 PMC5064969

[14] Salinas-Martínez AM, Amaya-Alemán MA, Arteaga-García JC, Núñez-Rocha GM, Garza-Elizondo ME. Eficiencia técnica de la atención al paciente con diabetes en el primer nivel. Salud Pública de México. 2009;51(1):48–58. 19180313 10.1590/s0036-36342009000100010

[15] Varela PS, de Andrade Martins G, Fávero LPL. Production efficiency and financing of public health: an analysis of small municipalities in the state of São Paulo-Brazil. Health Care Management Science. 2010;13(2):112–23. 20629414 10.1007/s10729-009-9114-y

[16] Sun D, Ahn H, Lievens T, Zeng W. Evaluation of the performance of national health systems in 2004–2011: an analysis of 173 countries. PloS One. 2017;12(3):e0173346 10.1371/journal.pone.0173346 28282397 PMC5345793

[17] Herrera S, Pang G. Efficiency of public spending in developing countries: an efficiency frontier approach Policy Research Working Paper 3645. Washington (D.C.): World Bank; 2005.

[18] Grigoli F, Kapsoli J. Waste not, want not: the efficiency of health expenditure in emerging and developing economies. Review of Development Economics. 2018;22(1):384–403.

[19] González E, Cárcaba A, Ventura J. Value efficiency analysis of health systems: does public financing play a role? Journal of Public Health. 2010;18(4):337–50.

[20] Jowett M, Brunal MP, Flores G, Cylus J. Spending targets for health: no magic number Health Financing Working Paper 1. Geneva: WHO; 2016.

[21] Greene W. Distinguishing between heterogeneity and inefficiency: stochastic frontier analysis of the World Health Organization′s panel data on national health care systems. Health Economics. 2004;13(10):959–80. 10.1002/hec.938 15455464

[22] de la Maisonneuve C, Moreno-Serra R, Murtin F, Oliveira Martins J. The role of policy and institutions on health spending. Health Economics. 2017;26(7):834–43. 10.1002/hec.3410 27683243

[23] de Cos PH, Moral-Benito E. Determinants of health-system efficiency: evidence from OECD countries. International Journal of Health Care Finance and Economics. 2014;14(1):69–93. 10.1007/s10754-013-9140-7 24398651

[24] Hadad S, Hadad Y, Simon-Tuval T. Determinants of healthcare system’s efficiency in OECD countries. The European Journal of Health Economics. 2013;14(2):253–65. 10.1007/s10198-011-0366-3 22146798

[25] United Nations. Sustainable Development Goals [cited 2017 Nov 17]. https://sustainabledevelopment.un.org/sdgs.

[26] Cylus J, Papanicolas I, Smith PC. How to make sense of health system efficiency comparisons? Copenhagen: World Health Organization, Regional Office for Europe; 2017. 29671993

[27] Jacobs R, Smith PC, Street A. Measuring efficiency in health care: analytic techniques and health policy. Cambridge: Cambridge University Press; 2006.

[28] Hollingsworth B, Smith P. Use of ratios in data envelopment analysis. Applied Economics Letters. 2003;10(11):733–5.

[29] World Health Organization. Tracking universal health coverage: first global monitoring report. Geneva: WHO; 2015.

[30] World Health Organization. Primary health care: now more than ever. Geneva: WHO; 2008.

[31] Moreno-Serra R, Smith PC. Does progress towards universal health coverage improve population health? The Lancet. 2012;380(9845):917–23. 10.1016/S0140-6736(12)61039-3 22959388

[32] Simar L, Wilson PW. Estimation and inference in two-stage, semi-parametric models of production processes. Journal of Econometrics. 2007;136(1):31–64.

[33] Afonso A, St. Aubyn M. Assessing health efficiency across countries with a two-step and bootstrap analysis. Applied Economics Letters. 2011;18(15):1427–30.

[34] Pérez-Cárceles MC, Gómez-Gallego JC, Gómez-Gallego M. Environmental factors affecting European and Central Asian health-systems’ bias-corrected efficiency. Applied Economics. 2018;50(32):3432–40.

[35] Moreno-Serra R, Smith PC. Broader health coverage is good for the nation′s health: evidence from country level panel data. Journal of the Royal Statistical Society: Series A (Statistics in Society). 2015;178(1):101–24. 10.1111/rssa.12048 PMC4280714 25598588

[36] Wagstaff A, Claeson M, Hecht RM, Gottret P, Fang Q. The Millennium Development Goals for health: rising to the challenges. Washington (D.C.): World Bank; 2004.

[37] Moreno-Serra R. The impact of cost-containment policies on health expenditure: evidence from recent OECD experiences. OECD Journal on Budgeting. 2014;12:1–29.

[38] Daraio C, Simar L, Wilson PW. Central limit theorems for conditional efficiency measures and tests of the ‘separability’condition in non-parametric, two-stage models of production. The Econometrics Journal. 2018;21(2):170–91.

[39] Montenegro Torres F. Costa Rica case study: primary health care achievements and challenges within the framework of the Social Health Insurance. Washington (D.C.): World Bank; 2013.

[40] Scrollini F, Ochoa UD. Perspectives on open government in Latin America. London: LSE, 2015.

[41] Bank World. Beyond the annual budget: global experience with medium-term expenditure frameworks. Washington (D.C.): World Bank; 2013.

[42] World Health Organization. Republic of Korea health system review. Manila: WHO Regional Office for the Western Pacific; 2015.

[43] Gottret PE, Schieber G. Health financing revisited: a practitioner′s guide. Washington (D.C.): World Bank; 2006.

[44] Dmytraczenko T, Almeida G. Toward universal health coverage and equity in Latin America and the Caribbean: evidence from selected countries. Washington (D.C.): World Bank; 2015.

