# Supplementary material for: Potential determinants of health system efficiency: Evidence from Latin America and the Caribbean
Source: PLoS One. 2019 May 10;14(5):e0216620. doi: 10.1371/journal.pone.0216620 (PMC6510473; doi:10.1371/journal.pone.0216620)
Supplement: S1 Appendix — (DOCX) [file pone.0216620.s001.docx]

**Table A1. Full list of sample countries: LAC, OECD and MICs.**

| No | Country | Code | Group |
| --- | --- | --- | --- |
| 1 | Argentina | ARG | LAC |
| 2 | Australia | AUS | OECD |
| 3 | Austria | AUT | OECD |
| 4 | Bahamas, The | BHS | LAC |
| 5 | Barbados | BRB | LAC |
| 6 | Belgium | BEL | OECD |
| 7 | Belize | BLZ | LAC |
| 8 | Bolivia | BOL | LAC |
| 9 | Botswana | BWA | MICs |
| 10 | Brazil | BRA | LAC |
| 11 | Cameroon | CMR | MICs |
| 12 | Canada | CAN | OECD |
| 13 | Chile | CHL | LAC |
| 14 | China | CHN | MICs |
| 15 | Colombia | COL | LAC |
| 16 | Costa Rica | CRI | LAC |
| 17 | Cuba | CUB | LAC |
| 18 | Czech Republic | CZE | OECD |
| 19 | Denmark | DNK | OECD |
| 20 | Dominican Republic | DOM | LAC |
| 21 | Ecuador | ECU | LAC |
| 22 | El Salvador | SLV | LAC |
| 23 | Estonia | EST | OECD |
| 24 | Finland | FIN | OECD |
| 25 | France | FRA | OECD |
| 26 | Germany | DEU | OECD |
| 27 | Ghana | GHA | MICs |
| 28 | Greece | GRC | OECD |
| 29 | Guatemala | GTM | LAC |
| 30 | Guyana | GUY | LAC |
| 31 | Haiti | HTI | LAC |
| 32 | Honduras | HND | LAC |
| 33 | Hungary | HUN | OECD |
| 34 | Iceland | ISL | OECD |
| 35 | India | IND | MICs |
| 36 | Indonesia | IDN | MICs |
| 37 | Ireland | IRL | OECD |
| 38 | Israel | ISR | OECD |
| 39 | Italy | ITA | OECD |
| 40 | Jamaica | JAM | LAC |
| 41 | Japan | JPN | OECD |
| 42 | Korea, Rep. | KOR | OECD |
| 43 | Luxembourg | LUX | OECD |
| 44 | Mexico | MEX | LAC |
| 45 | Netherlands | NLD | OECD |
| 46 | New Zealand | NZL | OECD |
| 47 | Nicaragua | NIC | LAC |
| 48 | Nigeria | NGA | MICs |
| 49 | Norway | NOR | OECD |
| 50 | Panama | PAN | LAC |
| 51 | Paraguay | PRY | LAC |
| 52 | Peru | PER | LAC |
| 53 | Poland | POL | OECD |
| 54 | Portugal | PRT | OECD |
| 55 | Russian Federation | RUS | MICs |
| 56 | Slovak Republic | SVK | OECD |
| 57 | Slovenia | SVN | OECD |
| 58 | South Africa | ZAF | MICs |
| 59 | Spain | ESP | OECD |
| 60 | Sri Lanka | LKA | MICs |
| 61 | Suriname | SUR | LAC |
| 62 | Sweden | SWE | OECD |
| 63 | Switzerland | CHE | OECD |
| 64 | Thailand | THA | MICs |
| 65 | Trinidad and Tobago | TTO | LAC |
| 66 | Turkey | TUR | OECD |
| 67 | United Kingdom | GBR | OECD |
| 68 | United States | USA | OECD |
| 69 | Uruguay | URY | LAC |
| 70 | Venezuela, RB | VEN | LAC |
| 71 | Vietnam | VNM | MICs |

**Table A2. Variables, period, sources and definition.**

| **Variables** | **Year of data** | **Source** | **Definition** |
| --- | --- | --- | --- |
| **Health outcomes** |  |  |  |
| Life expectancy at birth, total (years) | Average 2011-2015 | WB, WDI | Life expectancy at birth indicates the number of years a newborn infant would live if prevailing patterns of mortality at the time of its birth were to stay the same throughout its life. |
| Life expectancy at age 60, total (years) | Average 2011-2015 | WHO | The average number of years that a person of 60 years old could expect to live, if he or she were to pass through life exposed to the sex- and age-specific death rates prevailing at the time of his or her 60 years, for a specific year, in a given country, territory, or geographic area. |
| Mortality rate, under-5 (per 1,000 live births) | Average 2011-2015 | WB, WDI | Under-five mortality rate is the probability per 1,000 that a newborn baby will die before reaching age five, if subject to age-specific mortality rates of the specified year. |
| DALYs, All Causes, Age-standardized,  (per100,000), WHO | Average 2011-2015 | WHO | DALYs for a disease or health condition are calculated as the sum of the Years of Life Lost (YLL) due to premature mortality in the population and the Years Lost due to Disability (YLD) for people living with the health condition or its consequences |
| Skilled birth attendance (Perc. of total) | Average 2011-2015 | WB, WDI | Births attended by skilled health staff are the percentage of deliveries attended by personnel trained to give the necessary supervision, care, and advice to women during pregnancy, labor, and the postpartum period; to conduct deliveries on their own; and to care for newborns. |
| Immunization, DPT (Perc. of children ages 12-23  months) | Average 2011-2015 | WB, WDI | Child immunization measures the percentage of children ages 12-23 months who received vaccinations before 12 months or at any time before the survey. A child is considered adequately immunized against diphtheria, pertussis (or whooping cough), and tetanus (DPT) after receiving three doses of vaccine. |
| Skilled birth attendance ratio poorest/richest  quintile inequality | Latest available  from 1996-2015 |  | Ratio poorest/richest quintiles of births attended by skilled health staff. |
| Skilled birth attendance ratio rural/urban  inequality | Latest available  from 1996-2015 |  | Ratio rural/urban of births attended by skilled health staff. |
| **Inputs** |  |  |  |
| Health expenditure, public per capita PPP  (constant 2011 international USD $) | Average 2006-2010 | WB, WDI | Public health expenditure consists of recurrent and capital spending from government (central and local) budgets, external borrowings and grants (including donations from international agencies and nongovernmental organizations), and social (or compulsory) health insurance funds. Estimated by the authors from health expenditure per capita and health expenditure, pubic as percentage of percentage of total health expenditure. |
| Health expenditure per capita, PPP (constant  2011 international USD $) | Average 2006-2010 | WB, WDI | Total health expenditure is the sum of public and private health expenditures as a ratio of total population. It covers the provision of health services (preventive and curative), family planning activities, nutrition activities, and emergency aid designated for health but does not include provision of water and sanitation. Data are in international dollars converted using 2011 purchasing power parity (PPP) rates. |
| Pooled health expenditure per capita, PPP  (constant 2011 international USD $) | Average 2006-2010 | WB, WDI | Pooled health expenditure is the sum of public health expenditure and Voluntarily Health Insurance (VHI). VHI has been estimated for this report as a difference of private health expenditure and out-of-pocket health expenditure. Data are in international dollars converted using 2011 purchasing power parity (PPP) rates. |
| GDP per capita, PPP (constant 2011 international  USD) | Average 2006-2010 | WB, WDI | GDP per capita based on purchasing power parity (PPP). PPP GDP is gross domestic product converted to international dollars using purchasing power parity rates. An international dollar has the same purchasing power over GDP as the U.S. dollar has in the United States. GDP at purchaser's prices is the sum of gross value added by all resident producers in the economy plus any product taxes and minus any subsidies not included in the value of the products. It is calculated without making deductions for depreciation of fabricated assets or for depletion and degradation of natural resources. Data are in constant 2011 international dollars. |
| Population ages 65 and above (Perc. of total) | Average 2006-2010 | WB, WDI | Population ages 65 and above as a percentage of the total population. Population is based on the de facto definition of population, which counts all residents regardless of legal status or citizenship. |
| **Organization of healthcare delivery and financing** |  |  |  |
| Out-of-pocket health expenditure (Perc.) | Average 2006-2010 | WB, WDI | Out of pocket expenditure is any direct outlay by households, including gratuities and in-kind payments, to health practitioners and suppliers of pharmaceuticals, therapeutic appliances, and other goods and services whose primary intent is to contribute to the restoration or enhancement of the health status of individuals or population groups. It is a part of private health expenditure. |
| Hospital beds (per 1,000 people) | Average 2006-2010 | WB, WDI | Hospital beds include inpatient beds available in public, private, general, and specialized hospitals and rehabilitation centers. In most cases beds for both acute and chronic care are included. |
| **Quality of governance** |  |  |  |
| Control of Corruption | Average 2006-2010 | WB, WGI | Control of Corruption captures perceptions of the extent to which public power is exercised for private gain, including both petty and grand forms of corruption, as well as capture" of the state by elites and private interests. Estimate gives the country's score on the aggregate indicator in units of a standard normal distribution i.e. ranging from approximately -2.5 to 2.5." |
| Government Effectiveness | Average 2006-2010 | WB, WGI | Government Effectiveness captures perceptions of the quality of public services, the quality of the civil service and the degree of its independence from political pressures, the quality of policy formulation and implementation, and the credibility of the government's commitment to such policies. Estimate gives the country's score on the aggregate indicator, in units of a standard normal distribution, i.e. ranging from approximately -2.5 to 2.5. |
| Political Stability and Absence of  Violence/Terrorism | Average 2006-2010 | WB, WGI | Political Stability and Absence of Violence/Terrorism measures perceptions of the likelihood of political instability and/or politically-motivated violence, including terrorism. Estimate gives the country's score on the aggregate indicator, in units of a standard normal distribution, i.e. ranging from approximately -2.5 to 2.5. |
| Regulatory Quality | Average 2006-2010 | WB, WGI | Regulatory Quality captures perceptions of the ability of the government to formulate and implement sound policies and regulations that permit and promote private sector development. Estimate gives the country's score on the aggregate indicator, in units of a standard normal distribution, i.e. ranging from approximately -2.5 to 2.5. |
| Rule of Law | Average 2006-2010 | WB, WGI | Rule of Law captures perceptions of the extent to which agents have confidence in and abide by the rules of society, and in particular the quality of contract enforcement, property rights, the police, and the courts, as well as the likelihood of crime and violence. Estimate gives the country's score on the aggregate indicator, in units of a standard normal distribution, i.e. ranging from approximately -2.5 to 2.5. |
| Voice and Accountability | Average 2006-2010 | WB, WGI | Voice and Accountability captures perceptions of the extent to which a country's citizens are able to participate in selecting their government, as well as freedom of expression, freedom of association, and a free media. Estimate gives the country's score on the aggregate indicator, in units of a standard normal distribution, i.e. ranging from approximately -2.5 to 2.5. |
| Average governance quality | Average 2006-2010 | WB, WGI | Average of six governance quality indicators: Control of corruption, Government effectiveness, political stability and abnsence of violence/terrorism, Regulatory quality and Rule of law. |
| **Quality of health system institutions** |  |  |  |
| Medium term sectoral vision in line with the  government plan | 2013 | PRODEV Evaluation Tool (PET) | Composite index from 0 to 5 of the following indicators: a) There is a medium-term plan for the health sector, b) Civil Society participated in preparing the plan and c)The sectoral plan coincides with the government goals and targets |
| Results-based management in the production of  goods and services | 2013 | PRODEV Evaluation Tool (PET) | Composite index from 0 to 5 of the following indicators: a) There are annual and multi-annual targets for provision of good and services, b) There are offices responsible for achieving annual targets, c) Offices and programs sign performance contracts with the ministry/secretariat, d) Personnel remuneration and evaluation system incentivize results, e) An MfDR strategy is being implemented in the institution, f) Information is gathered on user opinions about good and services and g) Public consultation mechanisms are used to improve goods and services. |
| Sectoral information systems | 2013 | PRODEV Evaluation Tool (PET) | Composite index from 0 to 5 of the following indicators: a) There are information systems on the production of goods and services, b) There are information system on the quality of goods and services, c) There are indicators for the costs of goods and services, d) There are efficiency indicators for the coverage of goods and services and e) The information on management outcomes is available to citizens. |
| Average institutional quality | 2013 | PRODEV Evaluation Tool (PET) | Weighted average of the previous three indicators. |

Notes: WB, WDI = World Bank, World Development Indicators. WHO = World Health Organization. DALYs = Disability-adjusted life years. WB, WGI = World Bank, World Governance Indicators. IDB = Inter-American Development Bank.

**Table A3. Sample averages.**

| Country | Public health expenditure per capita (USD) | Total health expenditure per capita (USD) | Pooled health expenditure per capita (USD) | GDP per capita (USD) | Population 65+ | Life expectancy (years) | Life expectancy at age 60 (years) | Under-five mortality (per 1,000) | DALYs lost (per 100,000) |
| --- | --- | --- | --- | --- | --- | --- | --- | --- | --- |
| Argentina | 718.91 | 1172.28 | 896.24 | 17818.80 | 10.28 | 75.90 | 21.50 | 13.34 | 26808.20 |
| Australia | 2414.31 | 3568.67 | 2918.06 | 40767.79 | 13.19 | 82.09 | 25.00 | 4.12 | 17695.90 |
| Austria | 3145.53 | 4184.37 | 3485.63 | 42974.85 | 17.10 | 81.10 | 24.00 | 3.86 | 19763.40 |
| Bahamas, The | 774.33 | 1668.59 | 1189.76 | 24218.97 | 6.66 | 74.99 | 21.00 | 12.80 | 30459.30 |
| Barbados | 583.05 | 881.42 | 640.61 | 15746.56 | 12.10 | 75.25 | 23.00 | 13.70 | 26767.90 |
| Belgium | 2726.96 | 3554.96 | 2897.32 | 40689.27 | 17.14 | 80.54 | 23.50 | 4.26 | 19877.60 |
| Belize | 252.53 | 391.53 | 289.96 | 7907.28 | 3.60 | 69.95 | 21.00 | 17.50 | 31419.90 |
| Bolivia | 179.19 | 263.25 | 193.06 | 5137.10 | 5.72 | 67.66 | 19.00 | 41.50 | 44086.70 |
| Botswana | 468.03 | 680.72 | 649.73 | 12884.25 | 3.35 | 64.24 | 18.00 | 47.26 | 61512.80 |
| Brazil | 468.76 | 1074.74 | 731.47 | 13459.06 | 6.33 | 73.98 | 21.50 | 16.16 | 31631.70 |
| Cameroon | 29.34 | 122.29 | 34.25 | 2564.39 | 3.28 | 54.81 | 16.50 | 94.16 | 77368.50 |
| Canada | 2881.59 | 4099.43 | 3500.02 | 41017.11 | 13.66 | 81.66 | 25.00 | 5.16 | 18837.60 |
| Chile | 496.97 | 1125.25 | 717.53 | 18614.64 | 9.20 | 81.04 | 24.00 | 8.42 | 21332.60 |
| China | 176.65 | 354.87 | 211.71 | 7891.74 | 7.93 | 75.49 | 19.00 | 12.46 | 24811.00 |
| Colombia | 476.54 | 653.33 | 527.77 | 10422.95 | 5.56 | 73.72 | 24.50 | 16.92 | 27187.60 |
| Costa Rica | 743.64 | 1054.24 | 778.53 | 12261.65 | 7.03 | 79.14 | 23.00 | 9.96 | 23016.50 |
| Cuba | 1628.68 | 1715.76 | 1628.68 | 17449.71 | 11.89 | 79.21 | 22.00 | 5.72 | 25179.70 |
| Czech Republic | 1513.96 | 1795.94 | 1543.66 | 28129.87 | 14.73 | 78.10 | 21.50 | 3.66 | 22380.10 |
| Denmark | 3459.51 | 4079.86 | 3528.58 | 44311.75 | 15.99 | 80.18 | 23.00 | 3.70 | 20450.90 |
| Dominican Republic | 207.33 | 425.58 | 262.07 | 10451.26 | 5.91 | 73.23 | 22.00 | 32.12 | 29597.30 |
| Ecuador | 161.33 | 502.61 | 255.80 | 9107.74 | 5.87 | 75.55 | 22.00 | 23.02 | 29357.40 |
| El Salvador | 285.03 | 469.03 | 305.94 | 7464.21 | 6.98 | 72.36 | 22.00 | 18.02 | 36007.20 |
| Estonia | 930.24 | 1218.72 | 944.73 | 24087.69 | 17.27 | 76.73 | 21.50 | 3.52 | 26241.60 |
| Finland | 2320.27 | 3104.18 | 2513.57 | 40426.75 | 16.57 | 80.80 | 24.00 | 2.58 | 19843.50 |
| France | 2902.83 | 3744.25 | 3464.70 | 37018.99 | 16.78 | 82.17 | 25.00 | 4.34 | 19103.90 |
| Germany | 3045.46 | 4000.54 | 3446.81 | 40197.33 | 20.01 | 80.83 | 24.00 | 3.90 | 19223.70 |
| Ghana | 96.14 | 138.71 | 110.77 | 2845.96 | 3.49 | 61.06 | 17.00 | 66.64 | 58375.40 |
| Greece | 1786.97 | 2796.56 | 1882.61 | 31422.24 | 18.81 | 80.98 | 24.00 | 4.66 | 19627.40 |
| Guatemala | 149.94 | 429.15 | 190.49 | 6620.69 | 4.37 | 71.37 | 21.00 | 31.14 | 37078.80 |
| Guyana | 165.46 | 233.80 | 171.44 | 5407.04 | 4.28 | 66.27 | 15.00 | 40.60 | 55469.60 |
| Haiti | 20.49 | 95.96 | 59.35 | 1575.17 | 4.39 | 62.21 | 17.00 | 73.10 | 61716.60 |
| Honduras | 161.20 | 338.45 | 171.00 | 4297.14 | 4.30 | 72.85 | 22.00 | 21.88 | 33106.00 |
| Hungary | 1037.51 | 1550.02 | 1155.68 | 22760.50 | 16.32 | 75.34 | 20.00 | 6.18 | 28706.60 |
| Iceland | 2864.75 | 3497.16 | 2915.47 | 40819.12 | 11.92 | 82.35 | 24.50 | 2.14 | 17257.30 |
| India | 43.16 | 160.67 | 57.34 | 3879.39 | 4.99 | 67.47 | 17.00 | 52.26 | 47950.20 |
| Indonesia | 75.97 | 213.38 | 108.29 | 7790.32 | 4.92 | 68.61 | 18.00 | 29.36 | 36014.70 |
| Ireland | 2715.32 | 3686.90 | 3092.34 | 47210.98 | 10.87 | 80.95 | 24.00 | 3.84 | 19319.30 |
| Israel | 1244.62 | 2005.01 | 1476.50 | 28645.82 | 10.19 | 81.89 | 24.50 | 4.20 | 17719.10 |
| Italy | 2354.66 | 3065.94 | 2447.94 | 36854.61 | 20.03 | 82.45 | 25.00 | 3.70 | 16957.40 |
| Jamaica | 223.97 | 410.46 | 280.39 | 8765.88 | 8.25 | 75.38 | 21.00 | 16.68 | 32646.00 |
| Japan | 2359.74 | 2898.89 | 2458.38 | 34351.44 | 21.61 | 83.15 | 26.00 | 2.96 | 15699.60 |
| Korea, Rep. | 986.94 | 1777.17 | 1151.94 | 28520.74 | 10.33 | 81.51 | 24.00 | 3.70 | 17921.10 |
| Luxembourg | 5379.20 | 6229.84 | 5620.41 | 91960.83 | 14.14 | 81.60 | 25.00 | 2.06 | 18647.50 |
| Mexico | 411.10 | 892.92 | 444.65 | 15500.45 | 5.66 | 76.45 | 21.50 | 14.56 | 26763.10 |
| Netherlands | 3716.61 | 4324.74 | 4075.04 | 46175.82 | 14.84 | 81.23 | 24.00 | 4.04 | 18770.30 |
| New Zealand | 2463.69 | 2984.44 | 2635.58 | 32514.83 | 12.55 | 81.22 | 25.00 | 5.90 | 18741.80 |
| Nicaragua | 150.66 | 257.27 | 158.98 | 4007.26 | 4.56 | 74.36 | 21.00 | 23.60 | 33239.00 |
| Nigeria | 86.21 | 265.43 | 93.87 | 4714.11 | 2.75 | 52.26 | 16.00 | 116.86 | 84764.30 |
| Norway | 4328.05 | 5132.44 | 4367.87 | 64373.93 | 14.81 | 81.56 | 24.00 | 2.84 | 19614.60 |
| Panama | 660.35 | 949.88 | 709.58 | 14346.50 | 6.51 | 77.33 | 23.50 | 18.12 | 27475.70 |
| Paraguay | 191.77 | 493.03 | 224.46 | 6720.16 | 5.13 | 72.72 | 21.00 | 21.90 | 30861.90 |
| Peru | 225.04 | 417.05 | 253.82 | 8984.13 | 5.98 | 74.18 | 23.00 | 18.36 | 26910.50 |
| Poland | 858.16 | 1207.60 | 923.33 | 19923.76 | 13.36 | 76.92 | 21.00 | 5.28 | 25414.90 |
| Portugal | 1753.78 | 2573.84 | 1962.94 | 27375.68 | 18.08 | 80.57 | 24.00 | 3.74 | 19814.60 |
| Russian Federation | 668.22 | 1154.30 | 714.38 | 22600.96 | 13.43 | 70.19 | 17.00 | 10.42 | 39906.20 |
| Slovak Republic | 1208.66 | 1796.34 | 1351.12 | 23428.42 | 12.06 | 76.30 | 21.00 | 7.70 | 25991.00 |
| Slovenia | 1734.29 | 2358.61 | 2072.66 | 29072.64 | 16.23 | 80.23 | 23.00 | 2.84 | 21200.50 |
| South Africa | 410.89 | 891.95 | 816.40 | 11991.71 | 4.91 | 56.33 | 16.50 | 44.56 | 67513.80 |
| Spain | 2100.51 | 2830.63 | 2263.89 | 33953.61 | 16.90 | 82.76 | 25.00 | 4.30 | 16983.70 |
| Sri Lanka | 111.70 | 239.50 | 135.43 | 7738.43 | 7.10 | 74.60 | 20.00 | 10.20 | 28526.50 |
| Suriname | 390.24 | 781.17 | 677.57 | 13649.03 | 6.40 | 70.89 | 23.00 | 22.66 | 29900.60 |
| Sweden | 2902.10 | 3564.79 | 2989.16 | 42803.33 | 17.75 | 81.85 | 24.00 | 3.00 | 18307.60 |
| Switzerland | 3070.08 | 4887.72 | 3545.95 | 53930.09 | 16.42 | 82.76 | 25.00 | 4.16 | 17678.60 |
| Thailand | 514.68 | 624.14 | 558.40 | 12712.33 | 8.40 | 74.16 | 21.00 | 13.10 | 28992.80 |
| Trinidad and Tobago | 741.04 | 1443.32 | 866.55 | 30362.27 | 7.85 | 70.22 | 18.00 | 21.58 | 38359.70 |
| Turkey | 637.46 | 861.53 | 705.19 | 16219.42 | 6.84 | 74.77 | 21.00 | 15.50 | 29027.50 |
| United Kingdom | 2619.21 | 3187.32 | 2886.34 | 37118.64 | 16.05 | 80.98 | 24.00 | 4.60 | 20375.90 |
| United States | 3577.13 | 7742.58 | 6781.93 | 49984.98 | 12.66 | 78.79 | 23.00 | 6.88 | 22775.20 |
| Uruguay | 803.78 | 1298.32 | 1109.70 | 15221.37 | 13.79 | 76.76 | 22.00 | 11.08 | 24819.70 |
| Venezuela, RB | 338.83 | 807.61 | 393.87 | 16999.37 | 5.25 | 74.01 | 23.00 | 15.68 | 29409.70 |
| Vietnam | 94.34 | 227.61 | 110.68 | 4085.05 | 6.56 | 75.40 | 22.00 | 22.92 | 29225.60 |

**Table A3. Sample averages (continued).**

| Country | Skilled birth attendance (%) | DPT immuni-zation  (%) | Skilled birth attendance ratio  poorest/richest | Skilled birth attendance ratio  rural/urban | Out-of-pocket health expenditure (% total) | Hospital beds (per 1,000 people) | Government Effectiveness | Voice and Accoun-tability | Rule of Law |
| --- | --- | --- | --- | --- | --- | --- | --- | --- | --- |
| Argentina | 97.5 | 92.8 | 0.97 |  | 23.89 | 4.50 | -0.12 | 0.33 | -0.65 |
| Australia | 100.0 | 92.0 | 1.00 | 1.00 | 18.23 | 3.89 | 1.77 | 1.41 | 1.75 |
| Austria | 100.0 | 94.4 | 1.00 | 1.00 | 16.72 | 7.70 | 1.79 | 1.42 | 1.88 |
| Bahamas, The | 98.5 | 96.8 |  |  | 28.68 | 3.14 | 1.09 | 1.06 | 1.00 |
| Barbados | 99.0 | 92.0 | 1.00 | 1.02 | 27.36 | 6.90 | 1.47 | 1.16 | 1.15 |
| Belgium | 100.0 | 98.8 | 1.00 | 1.00 | 18.53 | 5.93 | 1.58 | 1.37 | 1.31 |
| Belize | 96.2 | 95.4 | 0.91 | 0.96 | 26.20 | 1.20 | -0.46 | 0.61 | -0.23 |
| Bolivia | 84.4 | 95.8 | 0.58 | 0.72 | 26.60 | 1.10 | -0.58 | -0.02 | -0.98 |
| Botswana |  | 95.0 |  | 0.91 | 4.65 | 2.00 | 0.53 | 0.46 | 0.64 |
| Brazil | 98.1 | 96.0 |  | 0.96 | 32.12 | 2.40 | -0.13 | 0.49 | -0.29 |
| Cameroon | 64.1 | 85.4 | 0.19 | 0.55 | 72.08 | 1.40 | -0.84 | -1.06 | -1.11 |
| Canada | 99.9 | 91.0 | 1.00 | 1.00 | 14.64 | 3.18 | 1.80 | 1.42 | 1.80 |
| Chile | 99.8 | 93.2 |  |  | 36.46 | 2.17 | 1.22 | 1.05 | 1.27 |
| China | 99.8 | 99.0 |  | 1.00 | 41.31 | 3.33 | 0.13 | -1.66 | -0.40 |
| Colombia | 99.2 | 89.6 | 0.86 | 0.88 | 19.23 | 1.00 | -0.10 | -0.17 | -0.44 |
| Costa Rica | 98.4 | 90.8 | 0.97 | 0.98 | 26.28 | 1.25 | 0.25 | 0.95 | 0.43 |
| Cuba | 99.8 | 98.6 |  | 1.01 | 5.30 | 5.52 | -0.33 | -1.73 | -0.67 |
| Czech Republic | 100.0 | 99.0 | 1.00 | 1.00 | 13.94 | 7.56 | 0.96 | 0.98 | 0.89 |
| Denmark | 100.0 | 93.2 | 1.00 | 1.00 | 13.54 | 3.53 | 2.24 | 1.58 | 1.95 |
| Dominican Republic | 98.0 | 85.6 | 1.00 | 0.98 | 38.65 | 1.15 | -0.62 | 0.11 | -0.69 |
| Ecuador | 92.1 | 84.6 | 0.82 | 0.85 | 49.16 | 1.53 | -0.84 | -0.28 | -1.15 |
| El Salvador | 98.8 | 91.6 | 0.93 | 0.96 | 34.80 | 0.90 | -0.12 | 0.07 | -0.75 |
| Estonia | 100.0 | 93.4 | 1.00 | 1.00 | 21.11 | 5.53 | 1.10 | 1.07 | 1.12 |
| Finland | 100.0 | 98.4 | 1.00 | 1.00 | 19.03 | 6.47 | 2.12 | 1.52 | 1.94 |
| France | 100.0 | 98.6 | 1.00 | 1.00 | 7.46 | 6.96 | 1.52 | 1.26 | 1.46 |
| Germany | 100.0 | 96.0 | 1.00 | 1.00 | 13.86 | 8.24 | 1.59 | 1.35 | 1.70 |
| Ghana | 71.1 | 91.8 | 0.44 | 0.64 | 20.31 | 0.90 | 0.04 | 0.44 | -0.05 |
| Greece | 100.0 | 99.0 | 1.00 | 1.00 | 32.62 | 4.80 | 0.60 | 0.91 | 0.75 |
| Guatemala | 62.8 | 83.2 | 0.39 | 0.66 | 55.63 | 0.63 | -0.62 | -0.25 | -1.10 |
| Guyana | 92.4 | 96.2 | 0.74 | 0.91 | 26.89 | 2.13 | -0.14 | 0.08 | -0.56 |
| Haiti | 37.3 | 62.2 | 0.13 | 0.42 | 38.63 | 1.30 | -1.41 | -0.69 | -1.38 |
| Honduras | 82.9 | 87.2 | 0.61 | 0.78 | 49.59 | 0.77 | -0.62 | -0.37 | -0.94 |
| Hungary | 100.0 | 99.0 | 1.00 | 1.00 | 25.42 | 7.26 | 0.73 | 0.96 | 0.86 |
| Iceland | 100.0 | 91.4 | 1.00 | 1.00 | 16.64 | 5.57 | 1.74 | 1.47 | 1.81 |
| India |  | 83.8 | 0.25 | 0.54 | 64.42 |  | 0.02 | 0.43 | 0.07 |
| Indonesia | 83.1 | 82.2 | 0.63 | 0.84 | 49.34 | 0.60 | -0.26 | -0.08 | -0.66 |
| Ireland | 100.0 | 95.4 | 1.00 | 1.00 | 16.11 | 4.64 | 1.46 | 1.40 | 1.72 |
| Israel | 100.0 | 94.0 | 1.00 | 1.00 | 25.92 | 5.10 | 1.29 | 0.66 | 0.85 |
| Italy | 100.0 | 95.4 | 1.00 | 1.00 | 20.18 | 3.68 | 0.35 | 1.03 | 0.39 |
| Jamaica | 99.1 | 92.8 | 0.96 | 0.98 | 31.57 | 1.83 | 0.25 | 0.54 | -0.46 |
| Japan | 100.0 | 96.4 | 1.00 | 1.00 | 15.24 | 13.83 | 1.49 | 0.97 | 1.32 |
| Korea, Rep. | 100.0 | 98.8 | 1.00 | 1.00 | 35.29 | 10.41 | 1.13 | 0.67 | 0.94 |
| Luxembourg | 100.0 | 99.0 | 1.00 | 1.00 | 9.79 | 5.53 | 1.65 | 1.54 | 1.78 |
| Mexico | 96.0 | 90.6 |  | 0.88 | 50.34 | 1.64 | 0.17 | 0.12 | -0.58 |
| Netherlands | 100.0 | 96.4 | 1.00 | 1.00 | 5.80 | 4.60 | 1.73 | 1.54 | 1.78 |
| New Zealand | 100.0 | 93.0 | 1.00 | 1.00 | 11.81 |  | 1.73 | 1.52 | 1.86 |
| Nicaragua | 88.0 | 98.0 | 0.42 | 0.81 | 38.24 | 0.90 | -0.92 | -0.36 | -0.80 |
| Nigeria | 43.4 | 48.2 | 0.10 | 0.42 | 65.07 |  | -1.06 | -0.77 | -1.11 |
| Norway | 100.0 | 94.2 | 1.00 | 1.00 | 14.89 | 3.62 | 1.90 | 1.59 | 1.93 |
| Panama | 92.5 | 81.0 | 0.72 | 0.79 | 25.60 | 2.27 | 0.13 | 0.57 | -0.16 |
| Paraguay | 95.8 | 88.4 |  |  | 53.88 | 1.30 | -0.89 | -0.19 | -0.98 |
| Peru | 87.2 | 90.4 | 0.65 | 0.73 | 39.31 | 1.43 | -0.41 | 0.06 | -0.71 |
| Poland | 100.0 | 98.6 | 1.00 | 1.00 | 23.56 | 6.27 | 0.49 | 0.91 | 0.50 |
| Portugal | 100.0 | 97.8 | 1.00 | 1.00 | 23.75 | 3.39 | 1.01 | 1.17 | 1.00 |
| Russian Federation |  | 97.0 |  |  | 36.81 | 9.70 | -0.40 | -0.89 | -0.87 |
| Slovak Republic | 100.0 | 97.8 | 1.00 | 1.00 | 24.92 | 6.63 | 0.85 | 0.90 | 0.51 |
| Slovenia | 100.0 | 95.4 | 1.00 | 1.00 | 12.13 | 4.67 | 1.06 | 1.04 | 0.96 |
| South Africa |  | 68.8 |  |  | 8.55 |  | 0.47 | 0.58 | 0.10 |
| Spain | 100.0 | 96.8 | 1.00 | 1.00 | 20.05 | 3.24 | 0.95 | 1.13 | 1.14 |
| Sri Lanka |  | 99.0 | 0.98 | 0.99 | 43.42 |  | -0.13 | -0.44 | 0.04 |
| Suriname |  | 86.0 | 0.86 | 0.88 | 13.24 | 3.03 | -0.14 | 0.45 | -0.24 |
| Sweden | 100.0 | 98.0 | 1.00 | 1.00 | 16.15 | 2.77 | 1.97 | 1.55 | 1.91 |
| Switzerland | 100.0 | 96.2 | 1.00 | 1.00 | 27.48 | 5.16 | 1.99 | 1.58 | 1.79 |
| Thailand | 99.6 | 99.0 | 0.98 | 1.00 | 10.61 | 2.10 | 0.30 | -0.56 | -0.13 |
| Trinidad and Tobago | 100.0 | 92.4 | 0.98 |  | 40.05 | 2.52 | 0.21 | 0.51 | -0.24 |
| Turkey | 97.4 | 96.8 | 0.91 | 0.93 | 18.31 | 2.58 | 0.27 | -0.10 | 0.07 |
| United Kingdom | 100.0 | 95.2 | 1.00 | 1.00 | 9.46 | 3.23 | 1.62 | 1.33 | 1.72 |
| United States | 100.0 | 94.8 | 1.00 | 1.00 | 12.43 | 3.06 | 1.58 | 1.10 | 1.59 |
| Uruguay | 98.9 | 94.8 |  | 1.00 | 14.39 | 2.33 | 0.54 | 1.06 | 0.59 |
| Venezuela, RB | 96.0 | 81.2 |  |  | 51.48 | 1.20 | -1.06 | -0.80 | -1.56 |
| Vietnam | 93.4 | 88.6 | 0.73 | 0.92 | 51.98 | 2.67 | -0.23 | -1.49 | -0.45 |

**Table A3. Sample averages (continued).**

| Country | Regulatory Quality | Political Stability and Absence of Violence/  Terrorism | Control of Corruption | | Average governance quality index | | Results-based management | | Sectoral information systems | | Medium-term sectoral vision | | Average health system institutional quality index | |  |
| --- | --- | --- | --- | --- | --- | --- | --- | --- | --- | --- | --- | --- | --- | --- | --- |
| Argentina | -0.73 | -0.05 | | -0.43 | | -0.28 | | 2.1 | | 1.9 | | 3.9 | | 2.5 | |
| Australia | 1.72 | 0.90 | | 2.05 | | 1.60 | |  | |  | |  | |  | |
| Austria | 1.58 | 1.19 | | 1.88 | | 1.62 | |  | |  | |  | |  | |
| Bahamas, The | 0.88 | 0.85 | | 1.37 | | 1.04 | | 1.2 | | 2.7 | | 2.0 | | 1.7 | |
| Barbados | 0.77 | 1.06 | | 1.35 | | 1.16 | | 1.9 | | 0.8 | | 3.8 | | 2.1 | |
| Belgium | 1.35 | 0.76 | | 1.36 | | 1.29 | |  | |  | |  | |  | |
| Belize | -0.44 | 0.10 | | -0.24 | | -0.11 | | 2.7 | | 1.1 | | 2.7 | | 2.3 | |
| Bolivia | -0.87 | -0.67 | | -0.46 | | -0.60 | |  | |  | |  | |  | |
| Botswana | 0.48 | 0.96 | | 0.96 | | 0.67 | |  | |  | |  | |  | |
| Brazil | 0.05 | -0.16 | | -0.08 | | -0.02 | | 3.1 | | 3.6 | | 4.6 | | 3.6 | |
| Cameroon | -0.79 | -0.47 | | -0.97 | | -0.87 | |  | |  | |  | |  | |
| Canada | 1.64 | 0.99 | | 2.02 | | 1.61 | |  | |  | |  | |  | |
| Chile | 1.49 | 0.53 | | 1.39 | | 1.16 | | 3.8 | | 3.7 | | 3.2 | | 3.6 | |
| China | -0.18 | -0.52 | | -0.56 | | -0.53 | |  | |  | |  | |  | |
| Colombia | 0.21 | -1.77 | | -0.24 | | -0.42 | | 4.0 | | 3.9 | | 4.3 | | 4.0 | |
| Costa Rica | 0.43 | 0.60 | | 0.50 | | 0.53 | | 1.3 | | 2.0 | | 4.6 | | 2.3 | |
| Cuba | -1.57 | 0.41 | | 0.33 | | -0.59 | |  | |  | |  | |  | |
| Czech Republic | 1.18 | 0.97 | | 0.28 | | 0.88 | |  | |  | |  | |  | |
| Denmark | 1.88 | 1.04 | | 2.50 | | 1.86 | |  | |  | |  | |  | |
| Dominican Republic | -0.20 | -0.06 | | -0.71 | | -0.36 | | 3.7 | | 2.9 | | 3.0 | | 3.3 | |
| Ecuador | -1.16 | -0.74 | | -0.85 | | -0.84 | | 2.0 | | 1.6 | | 4.1 | | 2.4 | |
| El Salvador | 0.25 | -0.01 | | -0.24 | | -0.13 | | 2.3 | | 2.3 | | 3.0 | | 2.5 | |
| Estonia | 1.38 | 0.59 | | 0.90 | | 1.03 | |  | |  | |  | |  | |
| Finland | 1.70 | 1.45 | | 2.39 | | 1.85 | |  | |  | |  | |  | |
| France | 1.26 | 0.56 | | 1.43 | | 1.25 | |  | |  | |  | |  | |
| Germany | 1.55 | 0.90 | | 1.74 | | 1.47 | |  | |  | |  | |  | |
| Ghana | 0.01 | 0.00 | | 0.02 | | 0.08 | |  | |  | |  | |  | |
| Greece | 0.81 | 0.22 | | 0.11 | | 0.57 | |  | |  | |  | |  | |
| Guatemala | -0.16 | -0.81 | | -0.60 | | -0.59 | | 1.6 | | 1.8 | | 2.0 | | 1.7 | |
| Guyana | -0.59 | -0.60 | | -0.57 | | -0.40 | | 2.1 | | 2.1 | | 2.8 | | 2.2 | |
| Haiti | -0.88 | -1.22 | | -1.23 | | -1.14 | | 1.0 | | 0.7 | | 0.6 | | 0.8 | |
| Honduras | -0.30 | -0.50 | | -0.80 | | -0.59 | | 2.7 | | 1.1 | | 4.8 | | 2.8 | |
| Hungary | 1.14 | 0.72 | | 0.43 | | 0.81 | |  | |  | |  | |  | |
| Iceland | 1.26 | 1.26 | | 2.19 | | 1.62 | |  | |  | |  | |  | |
| India | -0.31 | -1.17 | | -0.41 | | -0.23 | |  | |  | |  | |  | |
| Indonesia | -0.34 | -1.06 | | -0.70 | | -0.52 | |  | |  | |  | |  | |
| Ireland | 1.79 | 1.09 | | 1.74 | | 1.53 | |  | |  | |  | |  | |
| Israel | 1.13 | -1.36 | | 0.81 | | 0.56 | |  | |  | |  | |  | |
| Italy | 0.93 | 0.45 | | 0.23 | | 0.56 | |  | |  | |  | |  | |
| Jamaica | 0.29 | -0.30 | | -0.43 | | -0.02 | | 2.4 | | 1.6 | | 3.6 | | 2.5 | |
| Japan | 1.14 | 0.93 | | 1.36 | | 1.20 | |  | |  | |  | |  | |
| Korea, Rep. | 0.83 | 0.39 | | 0.41 | | 0.73 | |  | |  | |  | |  | |
| Luxembourg | 1.69 | 1.45 | | 2.00 | | 1.68 | |  | |  | |  | |  | |
| Mexico | 0.32 | -0.72 | | -0.29 | | -0.16 | | 3.3 | | 3.6 | | 4.2 | | 3.6 | |
| Netherlands | 1.74 | 0.87 | | 2.16 | | 1.64 | |  | |  | |  | |  | |
| New Zealand | 1.77 | 1.15 | | 2.38 | | 1.74 | |  | |  | |  | |  | |
| Nicaragua | -0.38 | -0.28 | | -0.74 | | -0.58 | | 2.4 | | 3.4 | | 4.1 | | 3.0 | |
| Nigeria | -0.80 | -2.01 | | -0.97 | | -1.12 | |  | |  | |  | |  | |
| Norway | 1.38 | 1.22 | | 2.02 | | 1.67 | |  | |  | |  | |  | |
| Panama | 0.41 | -0.05 | | -0.29 | | 0.10 | | 2.0 | | 2.6 | | 3.0 | | 2.4 | |
| Paraguay | -0.50 | -0.76 | | -1.00 | | -0.72 | | 1.6 | | 1.0 | | 3.0 | | 1.8 | |
| Peru | 0.33 | -0.93 | | -0.25 | | -0.32 | | 2.0 | | 1.6 | | 2.6 | | 2.1 | |
| Poland | 0.85 | 0.74 | | 0.30 | | 0.63 | |  | |  | |  | |  | |
| Portugal | 0.99 | 0.82 | | 1.00 | | 1.00 | |  | |  | |  | |  | |
| Russian Federation | -0.36 | -0.88 | | -1.00 | | -0.73 | |  | |  | |  | |  | |
| Slovak Republic | 1.07 | 0.95 | | 0.29 | | 0.76 | |  | |  | |  | |  | |
| Slovenia | 0.82 | 1.00 | | 0.96 | | 0.97 | |  | |  | |  | |  | |
| South Africa | 0.49 | 0.03 | | 0.21 | | 0.31 | |  | |  | |  | |  | |
| Spain | 1.19 | -0.33 | | 1.05 | | 0.85 | |  | |  | |  | |  | |
| Sri Lanka | -0.26 | -1.44 | | -0.24 | | -0.41 | |  | |  | |  | |  | |
| Suriname | -0.60 | 0.06 | | -0.25 | | -0.12 | | 2.3 | | 0.0 | | 4.0 | | 2.1 | |
| Sweden | 1.60 | 1.15 | | 2.26 | | 1.74 | |  | |  | |  | |  | |
| Switzerland | 1.59 | 1.25 | | 2.14 | | 1.72 | |  | |  | |  | |  | |
| Thailand | 0.22 | -1.28 | | -0.34 | | -0.30 | |  | |  | |  | |  | |
| Trinidad and Tobago | 0.59 | -0.14 | | -0.27 | | 0.11 | | 2.4 | | 1.6 | | 4.1 | | 2.6 | |
| Turkey | 0.30 | -0.84 | | 0.06 | | -0.04 | |  | |  | |  | |  | |
| United Kingdom | 1.76 | 0.43 | | 1.67 | | 1.42 | |  | |  | |  | |  | |
| United States | 1.51 | 0.46 | | 1.32 | | 1.26 | |  | |  | |  | |  | |
| Uruguay | 0.32 | 0.84 | | 1.17 | | 0.76 | | 1.9 | | 1.6 | | 2.7 | | 2.0 | |
| Venezuela, RB | -1.44 | -1.25 | | -1.09 | | -1.20 | |  | |  | |  | |  | |
| Vietnam | -0.60 | 0.21 | | -0.66 | | -0.54 | |  | |  | |  | |  | |

**Table A4. Average efficiency scores by output indicator and country.**

|  | Average efficiency scores by outcome | | | | | | | |  | Efficiency across  all DEA models | |  | Counting low and high performers across models | | | |  |
| --- | --- | --- | --- | --- | --- | --- | --- | --- | --- | --- | --- | --- | --- | --- | --- | --- | --- |
| COUNTRY | Life expectancy at birth (years) | Life expectancy at age 60 (years) | Under-five mortality (per 1000) | DALYs lost (per 100,000) | Skilled birth attendance (%) | DPT immunization (%) | Skilled birth attendance ratio poorest/richest | Skilled birth attendance ratio rural/urban |  | Average  efficiency | Ranking |  | Lowest to 25th | From 25 to 75th | 75th to highest | Number of estimations | Group |
| Argentina | 0.927 | 0.860 | 0.992 | 0.914 | 0.973 | 0.931 | 0.958 |  |  | 0.936 | 40 |  | 2 | 19 | 0 | 21 | LAC |
| Australia | 0.977 | 0.944 | 0.998 | 0.965 | 0.999 | 0.925 | 0.994 | 0.981 |  | 0.973 | 11 |  | 0 | 9 | 15 | 24 | OECD |
| Austria | 0.967 | 0.910 | 0.998 | 0.944 | 0.999 | 0.948 | 0.994 | 0.984 |  | 0.968 | 21 |  | 0 | 14 | 10 | 24 | OECD |
| Bahamas, The | 0.910 | 0.836 | 0.991 | 0.848 | 0.983 | 0.973 |  |  |  | 0.924 | 53 |  | 10 | 6 | 2 | 18 | LAC |
| Barbados | 0.932 | 0.924 | 0.992 | 0.931 | 0.987 | 0.919 | 0.974 | 0.989 |  | 0.956 | 32 |  | 2 | 19 | 3 | 24 | LAC |
| Belgium | 0.960 | 0.889 | 0.998 | 0.941 | 0.999 | 0.992 | 0.993 | 0.984 |  | 0.969 | 20 |  | 0 | 15 | 9 | 24 | OECD |
| Belize | 0.897 | 0.889 | 0.991 | 0.894 | 0.958 | 0.954 | 0.896 | 0.957 |  | 0.930 | 48 |  | 8 | 16 | 0 | 24 | LAC |
| Bolivia | 0.875 | 0.818 | 0.973 | 0.749 | 0.868 | 0.955 | 0.654 | 0.746 |  | 0.830 | 67 |  | 21 | 3 | 0 | 24 | LAC |
| Botswana | 0.799 | 0.716 | 0.960 | 0.492 |  | 0.953 |  | 0.894 |  | 0.802 | 68 |  | 15 | 3 | 0 | 18 | MICs |
| Brazil | 0.917 | 0.857 | 0.991 | 0.873 | 0.980 | 0.963 |  | 0.945 |  | 0.932 | 43 |  | 8 | 13 | 0 | 21 | LAC |
| Cameroon | 0.825 | 0.885 | 0.966 | 0.493 | 0.959 | 0.932 | 0.872 | 0.957 |  | 0.861 | 63 |  | 12 | 12 | 0 | 24 | MICs |
| Canada | 0.974 | 0.948 | 0.997 | 0.955 | 0.998 | 0.915 | 0.995 | 0.982 |  | 0.970 | 17 |  | 2 | 9 | 13 | 24 | OECD |
| Chile | 0.976 | 0.956 | 0.998 | 0.980 | 0.997 | 0.934 |  |  |  | 0.974 | 10 |  | 0 | 8 | 10 | 18 | LAC |
| China | 0.964 | 0.847 | 0.995 | 0.920 | 0.974 | 0.968 |  | 0.970 |  | 0.948 | 35 |  | 4 | 9 | 8 | 21 | MICs |
| Colombia | 0.922 | 0.961 | 0.991 | 0.935 | 0.991 | 0.899 | 0.846 | 0.871 |  | 0.927 | 49 |  | 11 | 10 | 3 | 24 | LAC |
| Costa Rica | 0.978 | 0.909 | 0.997 | 0.975 | 0.983 | 0.911 | 0.961 | 0.962 |  | 0.960 | 27 |  | 3 | 17 | 4 | 24 | LAC |
| Cuba | 0.969 | 0.864 | 0.998 | 0.923 | 0.996 | 0.989 |  | 0.985 |  | 0.960 | 25 |  | 1 | 12 | 8 | 21 | LAC |
| Czech Republic | 0.940 | 0.835 | 0.999 | 0.929 | 0.998 | 0.993 | 0.991 | 0.981 |  | 0.958 | 29 |  | 3 | 15 | 6 | 24 | OECD |
| Denmark | 0.957 | 0.873 | 0.998 | 0.937 | 0.999 | 0.937 | 0.994 | 0.983 |  | 0.960 | 26 |  | 0 | 13 | 11 | 24 | OECD |
| Dominican Republic | 0.940 | 0.941 | 0.976 | 0.915 | 0.975 | 0.855 | 0.971 | 0.974 |  | 0.944 | 37 |  | 6 | 15 | 3 | 24 | LAC |
| Ecuador | 0.976 | 0.951 | 0.985 | 0.929 | 0.927 | 0.841 | 0.801 | 0.848 |  | 0.907 | 54 |  | 15 | 3 | 6 | 24 | LAC |
| El Salvador | 0.925 | 0.921 | 0.991 | 0.836 | 0.989 | 0.914 | 0.922 | 0.962 |  | 0.933 | 42 |  | 7 | 17 | 0 | 24 | LAC |
| Estonia | 0.934 | 0.868 | 0.997 | 0.871 | 0.980 | 0.933 | 0.911 | 0.963 |  | 0.932 | 44 |  | 4 | 18 | 2 | 24 | OECD |
| Finland | 0.962 | 0.906 | 1.000 | 0.939 | 0.999 | 0.988 | 0.993 | 0.983 |  | 0.971 | 16 |  | 0 | 15 | 9 | 24 | OECD |
| France | 0.978 | 0.945 | 0.998 | 0.949 | 0.999 | 0.990 | 0.993 | 0.984 |  | 0.979 | 6 |  | 0 | 7 | 17 | 24 | OECD |
| Germany | 0.961 | 0.904 | 0.998 | 0.946 | 0.998 | 0.963 | 0.986 | 0.984 |  | 0.967 | 22 |  | 0 | 17 | 7 | 24 | OECD |
| Ghana | 0.838 | 0.797 | 0.968 | 0.676 | 0.905 | 0.942 | 0.801 | 0.850 |  | 0.847 | 65 |  | 16 | 8 | 0 | 24 | MICs |
| Greece | 0.971 | 0.925 | 0.998 | 0.954 | 0.997 | 0.985 | 0.984 | 0.982 |  | 0.975 | 8 |  | 0 | 15 | 9 | 24 | OECD |
| Guatemala | 0.923 | 0.910 | 0.980 | 0.832 | 0.638 | 0.826 | 0.404 | 0.668 |  | 0.772 | 69 |  | 20 | 4 | 0 | 24 | LAC |
| Guyana | 0.859 | 0.648 | 0.974 | 0.598 | 0.950 | 0.959 | 0.825 | 0.942 |  | 0.844 | 66 |  | 17 | 7 | 0 | 24 | LAC |
| Haiti | 0.933 | 0.886 | 0.992 | 0.813 | 0.947 | 0.920 | 0.774 | 0.924 |  | 0.899 | 55 |  | 12 | 12 | 0 | 24 | LAC |
| Honduras | 0.944 | 0.956 | 0.995 | 0.904 | 0.863 | 0.865 | 0.729 | 0.818 |  | 0.884 | 59 |  | 14 | 5 | 5 | 24 | LAC |
| Hungary | 0.923 | 0.808 | 0.996 | 0.880 | 0.996 | 0.980 | 0.976 | 0.980 |  | 0.942 | 38 |  | 7 | 15 | 2 | 24 | OECD |
| Iceland | 0.982 | 0.929 | 1.000 | 0.974 | 0.999 | 0.919 | 0.995 | 0.981 |  | 0.972 | 14 |  | 0 | 7 | 17 | 24 | OECD |
| India | 0.966 | 0.872 | 0.996 | 0.937 |  | 0.927 | 0.712 | 0.867 |  | 0.897 | 57 |  | 6 | 15 | 0 | 21 | MICs |
| Indonesia | 0.923 | 0.830 | 0.997 | 0.965 | 0.967 | 0.858 | 0.931 | 0.975 |  | 0.931 | 47 |  | 10 | 11 | 3 | 24 | MICs |
| Ireland | 0.965 | 0.910 | 0.998 | 0.949 | 0.999 | 0.959 | 0.995 | 0.981 |  | 0.970 | 19 |  | 0 | 14 | 10 | 24 | OECD |
| Israel | 0.990 | 0.962 | 0.999 | 0.982 | 0.999 | 0.945 | 0.992 | 0.979 |  | 0.981 | 4 |  | 0 | 10 | 14 | 24 | OECD |
| Italy | 0.977 | 0.935 | 0.998 | 0.965 | 0.997 | 0.955 | 0.983 | 0.982 |  | 0.974 | 9 |  | 0 | 13 | 11 | 24 | OECD |
| Jamaica | 0.967 | 0.901 | 0.992 | 0.872 | 0.986 | 0.922 | 0.940 | 0.976 |  | 0.945 | 36 |  | 3 | 20 | 1 | 24 | LAC |
| Japan | 0.972 | 0.953 | 0.997 | 0.931 | 0.985 | 0.953 | 0.914 | 0.964 |  | 0.959 | 28 |  | 3 | 12 | 9 | 24 | OECD |
| Korea, Rep. | 0.988 | 0.950 | 0.999 | 0.976 | 0.998 | 0.993 | 0.991 | 0.978 |  | 0.984 | 1 |  | 0 | 8 | 16 | 24 | OECD |
| Luxembourg | 0.974 | 0.951 | 1.000 | 0.959 | 0.999 | 0.995 | 0.995 | 0.982 |  | 0.982 | 2 |  | 0 | 1 | 23 | 24 | OECD |
| Mexico | 0.943 | 0.873 | 0.993 | 0.930 | 0.959 | 0.909 |  | 0.871 |  | 0.925 | 50 |  | 9 | 12 | 0 | 21 | LAC |
| Netherlands | 0.970 | 0.912 | 0.998 | 0.957 | 0.999 | 0.969 | 0.995 | 0.983 |  | 0.973 | 13 |  | 0 | 9 | 15 | 24 | OECD |
| New Zealand | 0.968 | 0.945 | 0.996 | 0.956 | 0.999 | 0.935 | 0.994 | 0.981 |  | 0.972 | 15 |  | 0 | 16 | 8 | 24 | OECD |
| Nicaragua | 0.966 | 0.916 | 0.993 | 0.911 | 0.930 | 0.964 | 0.517 | 0.857 |  | 0.882 | 60 |  | 10 | 13 | 1 | 24 | LAC |
| Nigeria | 0.686 | 0.709 | 0.905 | 0.216 | 0.479 | 0.502 | 0.134 | 0.470 |  | 0.513 | 71 |  | 24 | 0 | 0 | 24 | MICs |
| Norway | 0.974 | 0.913 | 0.999 | 0.948 | 0.999 | 0.947 | 0.995 | 0.983 |  | 0.970 | 18 |  | 0 | 9 | 15 | 24 | OECD |
| Panama | 0.952 | 0.933 | 0.988 | 0.918 | 0.923 | 0.813 | 0.711 | 0.770 |  | 0.876 | 61 |  | 15 | 9 | 0 | 24 | LAC |
| Paraguay | 0.938 | 0.903 | 0.990 | 0.914 | 0.967 | 0.883 |  |  |  | 0.933 | 41 |  | 7 | 11 | 0 | 18 | LAC |
| Peru | 0.949 | 0.977 | 0.990 | 0.951 | 0.870 | 0.904 | 0.641 | 0.723 |  | 0.875 | 62 |  | 14 | 7 | 3 | 24 | LAC |
| Poland | 0.942 | 0.843 | 0.999 | 0.928 | 0.997 | 0.986 | 0.985 | 0.977 |  | 0.957 | 30 |  | 3 | 15 | 6 | 24 | OECD |
| Portugal | 0.968 | 0.921 | 0.998 | 0.957 | 0.997 | 0.976 | 0.984 | 0.981 |  | 0.973 | 12 |  | 0 | 17 | 7 | 24 | OECD |
| Russian Federation | 0.863 | 0.691 | 0.994 | 0.752 |  | 0.974 |  |  |  | 0.855 | 64 |  | 9 | 3 | 3 | 15 | MICs |
| Slovak Republic | 0.923 | 0.824 | 0.995 | 0.901 | 0.998 | 0.982 | 0.991 | 0.979 |  | 0.949 | 34 |  | 5 | 16 | 3 | 24 | OECD |
| Slovenia | 0.961 | 0.884 | 0.999 | 0.940 | 0.998 | 0.956 | 0.991 | 0.982 |  | 0.964 | 24 |  | 0 | 20 | 4 | 24 | OECD |
| South Africa | 0.705 | 0.668 | 0.963 | 0.418 |  | 0.690 |  |  |  | 0.689 | 70 |  | 15 | 0 | 0 | 15 | MICs |
| Spain | 0.988 | 0.950 | 0.998 | 0.975 | 0.999 | 0.971 | 0.992 | 0.983 |  | 0.982 | 3 |  | 0 | 11 | 13 | 24 | OECD |
| Sri Lanka | 0.957 | 0.880 | 0.995 | 0.926 |  | 0.951 | 0.854 | 0.959 |  | 0.931 | 46 |  | 2 | 17 | 2 | 21 | MICs |
| Suriname | 0.882 | 0.940 | 0.985 | 0.896 |  | 0.863 | 0.848 | 0.869 |  | 0.898 | 56 |  | 15 | 5 | 1 | 21 | LAC |
| Sweden | 0.976 | 0.909 | 0.999 | 0.960 | 0.999 | 0.983 | 0.993 | 0.984 |  | 0.975 | 7 |  | 0 | 5 | 19 | 24 | OECD |
| Switzerland | 0.987 | 0.949 | 0.998 | 0.969 | 0.999 | 0.967 | 0.994 | 0.984 |  | 0.981 | 5 |  | 0 | 3 | 21 | 24 | OECD |
| Thailand | 0.920 | 0.841 | 0.994 | 0.905 | 0.994 | 0.993 | 0.970 | 0.984 |  | 0.950 | 33 |  | 4 | 15 | 5 | 24 | MICs |
| Trinidad and Tobago | 0.852 | 0.717 | 0.983 | 0.755 | 0.998 | 0.928 | 0.967 |  |  | 0.886 | 58 |  | 12 | 9 | 0 | 21 | LAC |
| Turkey | 0.916 | 0.836 | 0.990 | 0.895 | 0.973 | 0.972 | 0.900 | 0.912 |  | 0.924 | 52 |  | 13 | 10 | 1 | 24 | OECD |
| United Kingdom | 0.964 | 0.907 | 0.997 | 0.934 | 0.999 | 0.956 | 0.994 | 0.983 |  | 0.967 | 23 |  | 0 | 18 | 6 | 24 | OECD |
| United States | 0.941 | 0.874 | 0.995 | 0.910 | 0.999 | 0.953 | 0.995 | 0.981 |  | 0.956 | 31 |  | 0 | 16 | 8 | 24 | OECD |
| Uruguay | 0.942 | 0.880 | 0.994 | 0.902 | 0.978 | 0.942 |  | 0.958 |  | 0.942 | 39 |  | 3 | 18 | 0 | 21 | LAC |
| Venezuela, RB | 0.927 | 0.953 | 0.992 | 0.905 | 0.959 | 0.814 |  |  |  | 0.925 | 51 |  | 6 | 9 | 3 | 18 | LAC |
| Vietnam | 0.957 | 0.924 | 0.994 | 0.898 | 0.972 | 0.919 | 0.834 | 0.956 |  | 0.932 | 45 |  | 6 | 14 | 4 | 24 | MICs |
|  | Below 25th percentile | |  |  |  |  |  |  |  |  |  |  |  |  |  |  |  |
|  | Above 75th percentile | |  |  |  |  |  |  |  |  |  |  |  |  |  |  |  |

**Table A5. Further Simar-Wilson regression results of potential efficiency determinants, life expectancy at birth.**

|  |  |  |  |  |  |  |  |  |  |  |  |  |  |  |  |  |  |
| --- | --- | --- | --- | --- | --- | --- | --- | --- | --- | --- | --- | --- | --- | --- | --- | --- | --- |
|  | (1) | (2) | (3) | (4) | (5) | (6) | (7) | (8) | (9) | (10) | (11) | (12) | (13) | (14) | (15) | (16) | (17) |
| *Organization of healthcare delivery and financing* |  |  |  |  |  |  |  |  |  |  |  |  |  |  |  |  |  |
| Out-of-pocket health expenditure (Perc.) | 0.0004 |  |  |  |  |  |  |  |  |  |  |  |  |  |  |  | 0.001 |
|  | (0.001) |  |  |  |  |  |  |  |  |  |  |  |  |  |  |  | (0.001) |
| Hospital beds (per 1,000 people) |  | −0.0004 |  |  |  |  |  |  |  |  |  |  |  |  |  |  | −0.007 |
|  |  | (0.005) |  |  |  |  |  |  |  |  |  |  |  |  |  |  | (0.007) |
| *Quality of governance* |  |  |  |  |  |  |  |  |  |  |  |  |  |  |  |  |  |
| Government effectiveness |  |  | 0.001 |  |  |  |  |  |  |  |  |  |  | −0.034 |  |  |  |
|  |  |  | (0.011) |  |  |  |  |  |  |  |  |  |  | (0.030) |  |  |  |
| Voice and accountability |  |  |  | −0.007 |  |  |  |  |  |  |  |  |  | −0.041 |  |  |  |
|  |  |  |  | (0.012) |  |  |  |  |  |  |  |  |  | (0.026) |  |  |  |
| Rule of law |  |  |  |  | 0.004 |  |  |  |  |  |  |  |  | 0.011 |  |  |  |
|  |  |  |  |  | (0.011) |  |  |  |  |  |  |  |  | (0.044) |  |  |  |
| Regulatory quality |  |  |  |  |  | 0.005 |  |  |  |  |  |  |  | 0.033* |  |  |  |
|  |  |  |  |  |  | (0.011) |  |  |  |  |  |  |  | (0.018) |  |  |  |
| Political stability and absence of violence/terrorism |  |  |  |  |  |  | 0.007 |  |  |  |  |  |  | 0.02 |  |  |  |
|  |  |  |  |  |  |  | (0.011) |  |  |  |  |  |  | (0.020) |  |  |  |
| Control of corruption |  |  |  |  |  |  |  | 0.007 |  |  |  |  |  | 0.013 |  |  |  |
|  |  |  |  |  |  |  |  | (0.011) |  |  |  |  |  | (0.033) |  |  |  |
| Average governance quality |  |  |  |  |  |  |  |  | 0.004 |  |  |  |  |  |  | −0.001 | 0.016 |
|  |  |  |  |  |  |  |  |  | (0.013) |  |  |  |  |  |  | (0.013) | (0.015) |
| *Quality of health system institutions* |  |  |  |  |  |  |  |  |  |  |  |  |  |  |  |  |  |
| Medium term sectoral vision in line with the government plan |  |  |  |  |  |  |  |  |  | 0.005 |  |  |  |  | 0.006 | 0.006 | 0.008 |
|  |  |  |  |  |  |  |  |  |  | (0.008) |  |  |  |  | (0.008) | (0.008) | (0.008) |
| Results-based management in the production of goods and services |  |  |  |  |  |  |  |  |  |  | 0.001 |  |  |  | −0.013 | −0.013 | −0.012 |
|  |  |  |  |  |  |  |  |  |  |  | (0.010) |  |  |  | (0.013) | (0.013) | (0.012) |
| Sectoral information systems |  |  |  |  |  |  |  |  |  |  |  | 0.009 |  |  | 0.013 | 0.013 | 0.008 |
|  |  |  |  |  |  |  |  |  |  |  |  | (0.007) |  |  | (0.009) | (0.009) | (0.009) |
| Average institutional quality |  |  |  |  |  |  |  |  |  |  |  |  | 0.008 |  |  |  |  |
|  |  |  |  |  |  |  |  |  |  |  |  |  | (0.011) |  |  |  |  |
| Constant | 0.920*** | 0.934*** | 0.933*** | 0.934*** | 0.935*** | 0.934*** | 0.935*** | 0.934*** | 0.934*** | 0.917*** | 0.931*** | 0.916*** | 0.913*** | 0.950*** | 0.915*** | 0.915*** | 0.907*** |
|  | (0.021) | (0.013) | (0.009) | (0.009) | (0.011) | (0.009) | (0.009) | (0.009) | (0.009) | (0.028) | (0.025) | (0.016) | (0.028) | (0.018) | (0.028) | (0.028) | (0.042) |
| Observations | 27 | 27 | 27 | 27 | 27 | 27 | 27 | 27 | 27 | 24 | 24 | 24 | 24 | 27 | 24 | 24 | 24 |

Notes: Simar-Wilson models estimated with 1,000 bootstrap replications. *p<0.1, **p<0.05, ***p<0.01. Standard errors in parentheses.

**Table A6. Further Simar-Wilson regression results of potential efficiency determinants, life expectancy at age 60.**

|  |  |  |  |  |  |  |  |  |  |  |  |  |  |  |  |  |  |
| --- | --- | --- | --- | --- | --- | --- | --- | --- | --- | --- | --- | --- | --- | --- | --- | --- | --- |
|  | (1) | (2) | (3) | (4) | (5) | (6) | (7) | (8) | (9) | (10) | (11) | (12) | (13) | (14) | (15) | (16) | (17) |
| *Organization of healthcare delivery and financing* |  |  |  |  |  |  |  |  |  |  |  |  |  |  |  |  |  |
| Out-of-pocket health expenditure (Perc.) | 0.002 |  |  |  |  |  |  |  |  |  |  |  |  |  |  |  | 0.001 |
|  | (0.002) |  |  |  |  |  |  |  |  |  |  |  |  |  |  |  | (0.002) |
| Hospital beds (per 1,000 people) |  | −0.014 |  |  |  |  |  |  |  |  |  |  |  |  |  |  | −0.024 |
|  |  | (0.017) |  |  |  |  |  |  |  |  |  |  |  |  |  |  | (0.024) |
| *Quality of governance* |  |  |  |  |  |  |  |  |  |  |  |  |  |  |  |  |  |
| Government effectiveness |  |  | −0.022 |  |  |  |  |  |  |  |  |  |  | −0.165* |  |  |  |
|  |  |  | (0.221) |  |  |  |  |  |  |  |  |  |  | (0.094) |  |  |  |
| Voice and accountability |  |  |  | −0.015 |  |  |  |  |  |  |  |  |  | −0.019 |  |  |  |
|  |  |  |  | (0.045) |  |  |  |  |  |  |  |  |  | (0.073) |  |  |  |
| Rule of law |  |  |  |  | −0.008 |  |  |  |  |  |  |  |  | 0.004 |  |  |  |
|  |  |  |  |  | (0.038) |  |  |  |  |  |  |  |  | (0.134) |  |  |  |
| Regulatory quality |  |  |  |  |  | 0.011 |  |  |  |  |  |  |  | 0.081 |  |  |  |
|  |  |  |  |  |  | (0.038) |  |  |  |  |  |  |  | (0.056) |  |  |  |
| Political stability and absence of violence/terrorism |  |  |  |  |  |  | −0.019 |  |  |  |  |  |  | −0.023 |  |  |  |
|  |  |  |  |  |  |  | (0.050) |  |  |  |  |  |  | (0.059) |  |  |  |
| Control of corruption |  |  |  |  |  |  |  | −0.003 |  |  |  |  |  | 0.106 |  |  |  |
|  |  |  |  |  |  |  |  | (0.038) |  |  |  |  |  | (0.102) |  |  |  |
| Average governance quality |  |  |  |  |  |  |  |  | −0.011 |  |  |  |  |  |  | −0.005 | 0.04 |
|  |  |  |  |  |  |  |  |  | (0.057) |  |  |  |  |  |  | (0.054) | (0.055) |
| *Quality of health system institutions* |  |  |  |  |  |  |  |  |  |  |  |  |  |  |  |  |  |
| Medium term sectoral vision in line with the government plan |  |  |  |  |  |  |  |  |  | 0.015 |  |  |  |  | 0.0001 | 0.001 | 0.006 |
|  |  |  |  |  |  |  |  |  |  | (0.036) |  |  |  |  | (0.061) | (0.034) | (0.028) |
| Results-based management in the production of goods and services |  |  |  |  |  |  |  |  |  |  | 0.038 |  |  |  | 0.053 | 0.052 | 0.051 |
|  |  |  |  |  |  |  |  |  |  |  | (0.061) |  |  |  | (0.084) | (0.062) | (0.045) |
| Sectoral information systems |  |  |  |  |  |  |  |  |  |  |  | 0.002 |  |  | −0.022 | −0.021 | −0.035 |
|  |  |  |  |  |  |  |  |  |  |  |  | (0.398) |  |  | (0.043) | (0.051) | (0.031) |
| Average institutional quality |  |  |  |  |  |  |  |  |  |  |  |  | 0.033 |  |  |  |  |
|  |  |  |  |  |  |  |  |  |  |  |  |  | (0.047) |  |  |  |  |
| Constant | 0.869*** | 0.960*** | 0.929 | 0.935*** | 0.930*** | 0.934*** | 0.928*** | 0.933*** | 0.931*** | 0.895*** | 0.855*** | 0.943 | 0.862*** | 0.927*** | 0.862*** | 0.860*** | 0.878*** |
|  | (0.085) | (0.094) | (3.462) | (0.121) | (0.072) | (0.078) | (0.122) | (0.076) | (0.141) | (0.164) | (0.172) | (3.143) | (0.153) | (0.060) | (0.166) | (0.140) | (0.145) |
| Observations | 27 | 27 | 27 | 27 | 27 | 27 | 27 | 27 | 27 | 24 | 24 | 24 | 24 | 27 | 24 | 24 | 24 |

Notes: Simar-Wilson models estimated with 1,000 bootstrap replications. *p<0.1, **p<0.05, ***p<0.01. Standard errors in parentheses.

**Table A7. Further Simar-Wilson regression results of potential efficiency determinants, under-five mortality.**

|  |  |  |  |  |  |  |  |  |  |  |  |  |  |  |  |  |  |
| --- | --- | --- | --- | --- | --- | --- | --- | --- | --- | --- | --- | --- | --- | --- | --- | --- | --- |
|  | (1) | (2) | (3) | (4) | (5) | (6) | (7) | (8) | (9) | (10) | (11) | (12) | (13) | (14) | (15) | (16) | (17) |
| *Organization of healthcare delivery and financing* |  |  |  |  |  |  |  |  |  |  |  |  |  |  |  |  |  |
| Out-of-pocket health expenditure (Perc.) | −0.001 |  |  |  |  |  |  |  |  |  |  |  |  |  |  |  | -0.001 |
|  | (0.001) |  |  |  |  |  |  |  |  |  |  |  |  |  |  |  | (0.001) |
| Hospital beds (per 1,000 people) |  | 0.002 |  |  |  |  |  |  |  |  |  |  |  |  |  |  | −0.002 |
|  |  | (0.002) |  |  |  |  |  |  |  |  |  |  |  |  |  |  | (0.001) |
| *Quality of governance* |  |  |  |  |  |  |  |  |  |  |  |  |  |  |  |  |  |
| Government effectiveness |  |  | 0.004 |  |  |  |  |  |  |  |  |  |  | −0.005 |  |  |  |
|  |  |  | (0.003) |  |  |  |  |  |  |  |  |  |  | (0.007) |  |  |  |
| Voice and accountability |  |  |  | 0.001 |  |  |  |  |  |  |  |  |  | −0.014* |  |  |  |
|  |  |  |  | (0.003) |  |  |  |  |  |  |  |  |  | (0.007) |  |  |  |
| Rule of law |  |  |  |  | 0.005 |  |  |  |  |  |  |  |  | 0.009 |  |  |  |
|  |  |  |  |  | (0.004) |  |  |  |  |  |  |  |  | (0.010) |  |  |  |
| Regulatory quality |  |  |  |  |  | 0.003 |  |  |  |  |  |  |  | 0.005 |  |  |  |
|  |  |  |  |  |  | (0.003) |  |  |  |  |  |  |  | (0.004) |  |  |  |
| Political stability and absence of violence/terrorism |  |  |  |  |  |  | 0.004 |  |  |  |  |  |  | 0.003 |  |  |  |
|  |  |  |  |  |  |  | (0.003) |  |  |  |  |  |  | (0.004) |  |  |  |
| Control of corruption |  |  |  |  |  |  |  | 0.006* |  |  |  |  |  | 0.004 |  |  |  |
|  |  |  |  |  |  |  |  | (0.003) |  |  |  |  |  | (0.008) |  |  |  |
| Average governance quality |  |  |  |  |  |  |  |  | 0.005 |  |  |  |  |  |  | 0.004* | 0.006* |
|  |  |  |  |  |  |  |  |  | (0.004) |  |  |  |  |  |  | (0.002) | (0.003) |
| *Quality of health system institutions* |  |  |  |  |  |  |  |  |  |  |  |  |  |  |  |  |  |
| Medium term sectoral vision in line with the government plan |  |  |  |  |  |  |  |  |  | 0.002 |  |  |  |  | 0.002 | 0.002 | 0.002 |
|  |  |  |  |  |  |  |  |  |  | (0.002) |  |  |  |  | (0.002) | (0.002) | (0.002) |
| Results-based management in the production of goods and services |  |  |  |  |  |  |  |  |  |  | 0.00005 |  |  |  | −0.003 | −0.002 | −0.003 |
|  |  |  |  |  |  |  |  |  |  |  | (0.002) |  |  |  | (0.003) | (0.003) | (0.003) |
| Sectoral information systems |  |  |  |  |  |  |  |  |  |  |  | 0.001 |  |  | 0.002 | 0.001 | 0.0005 |
|  |  |  |  |  |  |  |  |  |  |  |  | (0.002) |  |  | (0.002) | (0.002) | (0.002) |
| Average institutional quality |  |  |  |  |  |  |  |  |  |  |  |  | 0.001 |  |  |  |  |
|  |  |  |  |  |  |  |  |  |  |  |  |  | (0.002) |  |  |  |  |
| Constant | 0.995*** | 0.988*** | 0.992*** | 0.991*** | 0.993*** | 0.992*** | 0.992*** | 0.992*** | 0.992*** | 0.985*** | 0.990*** | 0.988*** | 0.987*** | 0.999*** | 0.986*** | 0.988*** | 0.992*** |
|  | (0.007) | (0.005) | (0.003) | (0.004) | (0.006) | (0.004) | (0.003) | (0.003) | (0.004) | (0.005) | (0.005) | (0.004) | (0.006) | (0.005) | (0.006) | (0.006) | (0.008) |
| Observations | 27 | 27 | 27 | 27 | 27 | 27 | 27 | 27 | 27 | 24 | 24 | 24 | 24 | 27 | 24 | 24 | 24 |
| Notes: Simar-Wilson models estimated with 1,000 bootstrap replications. *p<0.1, **p<0.05, ***p<0.01. Standard errors in parentheses. For specifications (1) and (17), the very small coefficient and standard error for out-of-pocket health expenditure are rounded to three decimals. | | | | | | | | | | | | | | | | | |

**Table A8. Further Simar-Wilson regression results of potential efficiency determinants, DALYs lost.**

|  |  |  |  |  |  |  |  |  |  |  |  |  |  |  |  |  |  |
| --- | --- | --- | --- | --- | --- | --- | --- | --- | --- | --- | --- | --- | --- | --- | --- | --- | --- |
|  | (1) | (2) | (3) | (4) | (5) | (6) | (7) | (8) | (9) | (10) | (11) | (12) | (13) | (14) | (15) | (16) | (17) |
| *Organization of healthcare delivery and financing* |  |  |  |  |  |  |  |  |  |  |  |  |  |  |  |  |  |
| Out-of-pocket health expenditure (Perc.) | 0.0002 |  |  |  |  |  |  |  |  |  |  |  |  |  |  |  | 0.002 |
|  | (0.003) |  |  |  |  |  |  |  |  |  |  |  |  |  |  |  | (0.002) |
| Hospital beds (per 1,000 people) |  | 0.011 |  |  |  |  |  |  |  |  |  |  |  |  |  |  | −0.013 |
|  |  | (0.184) |  |  |  |  |  |  |  |  |  |  |  |  |  |  | (0.023) |
| *Quality of governance* |  |  |  |  |  |  |  |  |  |  |  |  |  |  |  |  |  |
| Government effectiveness |  |  | 0.032 |  |  |  |  |  |  |  |  |  |  | −0.144 |  |  |  |
|  |  |  | (0.052) |  |  |  |  |  |  |  |  |  |  | (0.104) |  |  |  |
| Voice and accountability |  |  |  | 0.019 |  |  |  |  |  |  |  |  |  | −0.060 |  |  |  |
|  |  |  |  | (0.061) |  |  |  |  |  |  |  |  |  | (0.097) |  |  |  |
| Rule of law |  |  |  |  | 0.049 |  |  |  |  |  |  |  |  | 0.097 |  |  |  |
|  |  |  |  |  | (0.047) |  |  |  |  |  |  |  |  | (0.152) |  |  |  |
| Regulatory quality |  |  |  |  |  | 0.039 |  |  |  |  |  |  |  | 0.062 |  |  |  |
|  |  |  |  |  |  | (0.329) |  |  |  |  |  |  |  | (0.063) |  |  |  |
| Political stability and absence of violence/terrorism |  |  |  |  |  |  | 0.039 |  |  |  |  |  |  | 0.003 |  |  |  |
|  |  |  |  |  |  |  | (0.053) |  |  |  |  |  |  | (0.064) |  |  |  |
| Control of corruption |  |  |  |  |  |  |  | 0.053 |  |  |  |  |  | 0.073 |  |  |  |
|  |  |  |  |  |  |  |  | (0.051) |  |  |  |  |  | (0.115) |  |  |  |
| Average governance quality |  |  |  |  |  |  |  |  | 0.05 |  |  |  |  |  |  | 0.04 | 0.072 |
|  |  |  |  |  |  |  |  |  | (0.050) |  |  |  |  |  |  | (0.042) | (0.056) |
| *Quality of health system institutions* |  |  |  |  |  |  |  |  |  |  |  |  |  |  |  |  |  |
| Medium term sectoral vision in line with the government plan |  |  |  |  |  |  |  |  |  | 0.042 |  |  |  |  | 0.034 | 0.028 | 0.03 |
|  |  |  |  |  |  |  |  |  |  | (0.031) |  |  |  |  | (0.031) | (0.028) | (0.028) |
| Results-based management in the production of goods and services |  |  |  |  |  |  |  |  |  |  | 0.047 |  |  |  | 0.016 | 0.021 | 0.023 |
|  |  |  |  |  |  |  |  |  |  |  | (0.645) |  |  |  | (0.048) | (0.047) | (0.043) |
| Sectoral information systems |  |  |  |  |  |  |  |  |  |  |  | 0.022 |  |  | 0.005 | −0.002 | −0.011 |
|  |  |  |  |  |  |  |  |  |  |  |  | (0.041) |  |  | (0.032) | (0.032) | (0.029) |
| Average institutional quality |  |  |  |  |  |  |  |  |  |  |  |  | 0.062 |  |  |  |  |
|  |  |  |  |  |  |  |  |  |  |  |  |  | (0.05) |  |  |  |  |
| Constant | 0.921 | 0.903 | 0.930*** | 0.923*** | 0.943*** | 0.928 | 0.933*** | 0.932*** | 0.931*** | 0.783*** | 0.821 | 0.886*** | 0.772*** | 0.965*** | 0.761*** | 0.783*** | 0.765*** |
|  | (0.625) | (1.382) | (0.103) | (0.177) | (0.069) | (2.597) | (0.139) | (0.078) | (0.067) | (0.097) | (2.427) | (0.102) | (0.124) | (0.073) | (0.098) | (0.089) | (0.149) |
| Observations | 27 | 27 | 27 | 27 | 27 | 27 | 27 | 27 | 27 | 24 | 24 | 24 | 24 | 27 | 24 | 24 | 24 |

Notes: Simar-Wilson models estimated with 1,000 bootstrap replications. *p<0.1, **p<0.05, ***p<0.01. Standard errors in parentheses.

**Table A9. Further Simar-Wilson regression results of potential efficiency determinants, skilled birth attendance.**

|  |  |  |  |  |  |  |  |
| --- | --- | --- | --- | --- | --- | --- | --- |
|  | (1) | (5) | (9) | (11) | (14) | (16) | (17) |
| *Organization of healthcare delivery and financing* |  |  |  |  |  |  |  |
| Out-of-pocket health expenditure (Perc.) | −0.126 |  |  |  |  |  | −0.014 |
|  | (0.110) |  |  |  |  |  | (0.010) |
| Hospital beds (per 1,000 people) |  |  |  |  |  |  | 0.206 |
|  |  |  |  |  |  |  | (0.130) |
| *Quality of governance* |  |  |  |  |  |  |  |
| Government effectiveness |  |  |  |  | 0.614 |  |  |
|  |  |  |  |  | (0.430) |  |  |
| Voice and accountability |  |  |  |  | −0.992* |  |  |
|  |  |  |  |  | (0.570) |  |  |
| Rule of law |  | 2.602 |  |  | 1.991* |  |  |
|  |  | (2.060) |  |  | (1.020) |  |  |
| Regulatory quality |  |  |  |  | −0.466 |  |  |
|  |  |  |  |  | (0.310) |  |  |
| Political stability and absence of violence/terrorism |  |  |  |  | −0.033 |  |  |
|  |  |  |  |  | (0.210) |  |  |
| Control of corruption |  |  |  |  | −0.701 |  |  |
|  |  |  |  |  | (0.500) |  |  |
| Average governance quality |  |  | 11.795*** |  |  | 2.845*** | 0.08 |
|  |  |  | (2.983) |  |  | (0.590) | (0.160) |
| *Quality of health system institutions* |  |  |  |  |  |  |  |
| Medium term sectoral vision in line with the government plan |  |  |  |  |  | −0.235 | 0.014 |
|  |  |  |  |  |  | (0.240) | (0.060) |
| Results-based management in the production of goods and services |  |  |  | 10.031*** |  | 1.258*** | 0.143 |
|  |  |  |  | (2.510) |  | (0.360) | (0.130) |
| Sectoral information systems |  |  |  |  |  | −0.491** | −0.036 |
|  |  |  |  |  |  | (0.240) | (0.090) |
| Constant | 8.465 | 5.368 | 19.587*** | 0.897 | 2.615*** | 3.780*** | 1.242** |
|  | (7.860) | (4.670) | (7.421) | (7.650) | (0.920) | (1.140) | (0.510) |
| Observations | 26 | 26 | 26 | 23 | 26 | 23 | 23 |
| Notes: Simar-Wilson models estimated with 1,000 bootstrap replications. *p<0.1, **p<0.05, ***p<0.01. Standard errors in parentheses. Results for some specifications could not be obtained because the corresponding Simar-Wilson regressions failed to reach convergence. The reason behind the lack of statistical convergence for these models seems to be relatively limited cross-country variation in the estimated skilled birth attendance efficiency scores, which is compounded in some cases by less variation in specific efficiency determinant indicators. | | | | | | | |
|  | | | | | | | |

**Table A10. Further Simar-Wilson regression results of potential efficiency determinants, DPT immunization.**

|  |  |  |  |  |  |  |  |  |  |  |  |  |  |  |  |  |  |
| --- | --- | --- | --- | --- | --- | --- | --- | --- | --- | --- | --- | --- | --- | --- | --- | --- | --- |
|  | (1) | (2) | (3) | (4) | (5) | (6) | (7) | (8) | (9) | (10) | (11) | (12) | (13) | (14) | (15) | (16) | (17) |
| *Organization of healthcare delivery and financing* |  |  |  |  |  |  |  |  |  |  |  |  |  |  |  |  |  |
| Out-of-pocket health expenditure (Perc.) | −0.002*** |  |  |  |  |  |  |  |  |  |  |  |  |  |  |  | −0.001 |
|  | (0.001) |  |  |  |  |  |  |  |  |  |  |  |  |  |  |  | (0.001) |
| Hospital beds (per 1,000 people) |  | 0.016* |  |  |  |  |  |  |  |  |  |  |  |  |  |  | 0.003 |
|  |  | (0.010) |  |  |  |  |  |  |  |  |  |  |  |  |  |  | (0.009) |
| *Quality of governance* |  |  |  |  |  |  |  |  |  |  |  |  |  |  |  |  |  |
| Government effectiveness |  |  | 0.029 |  |  |  |  |  |  |  |  |  |  | −0.017 |  |  |  |
|  |  |  | (0.019) |  |  |  |  |  |  |  |  |  |  | (0.044) |  |  |  |
| Voice and accountability |  |  |  | 0.013 |  |  |  |  |  |  |  |  |  | −0.030 |  |  |  |
|  |  |  |  | (0.018) |  |  |  |  |  |  |  |  |  | (0.042) |  |  |  |
| Rule of law |  |  |  |  | 0.033* |  |  |  |  |  |  |  |  | 0.026 |  |  |  |
|  |  |  |  |  | (0.018) |  |  |  |  |  |  |  |  | (0.064) |  |  |  |
| Regulatory quality |  |  |  |  |  | 0.012 |  |  |  |  |  |  |  | −0.012 |  |  |  |
|  |  |  |  |  |  | (0.018) |  |  |  |  |  |  |  | (0.027) |  |  |  |
| Political stability and absence of violence/terrorism |  |  |  |  |  |  | 0.035* |  |  |  |  |  |  | 0.011 |  |  |  |
|  |  |  |  |  |  |  | (0.018) |  |  |  |  |  |  | (0.026) |  |  |  |
| Control of corruption |  |  |  |  |  |  |  | 0.039** |  |  |  |  |  | 0.047 |  |  |  |
|  |  |  |  |  |  |  |  | (0.018) |  |  |  |  |  | (0.051) |  |  |  |
| Average governance quality |  |  |  |  |  |  |  |  | 0.034 |  |  |  |  |  |  | 0.028* | 0.013 |
|  |  |  |  |  |  |  |  |  | (0.021) |  |  |  |  |  |  | (0.017) | (0.020) |
| *Quality of health system institutions* |  |  |  |  |  |  |  |  |  |  |  |  |  |  |  |  |  |
| Medium term sectoral vision in line with the government plan |  |  |  |  |  |  |  |  |  | −0.002 |  |  |  |  | −0.001 | −0.004 | −0.004 |
|  |  |  |  |  |  |  |  |  |  | (0.011) |  |  |  |  | (0.011) | (0.011) | (0.010) |
| Results-based management in the production of goods and services |  |  |  |  |  |  |  |  |  |  | 0.001 |  |  |  | −0.012 | −0.009 | −0.011 |
|  |  |  |  |  |  |  |  |  |  |  | (0.014) |  |  |  | (0.018) | (0.016) | (0.016) |
| Sectoral information systems |  |  |  |  |  |  |  |  |  |  |  | 0.011 |  |  | 0.016 | 0.013 | 0.016 |
|  |  |  |  |  |  |  |  |  |  |  |  | (0.010) |  |  | (0.012) | (0.011) | (0.011) |
| Average institutional quality |  |  |  |  |  |  |  |  |  |  |  |  | 0.005 |  |  |  |  |
|  |  |  |  |  |  |  |  |  |  |  |  |  | (0.015) |  |  |  |  |
| Constant | 0.992*** | 0.883*** | 0.921*** | 0.915*** | 0.930*** | 0.918*** | 0.924*** | 0.923*** | 0.922*** | 0.917*** | 0.910*** | 0.889*** | 0.899*** | 0.939*** | 0.909*** | 0.920*** | 0.945*** |
|  | (0.034) | (0.021) | (0.015) | (0.014) | (0.017) | (0.016) | (0.014) | (0.014) | (0.014) | (0.038) | (0.033) | (0.022) | (0.037) | (0.028) | (0.039) | (0.037) | (0.053) |
| Observations | 27 | 27 | 27 | 27 | 27 | 27 | 27 | 27 | 27 | 24 | 24 | 24 | 24 | 27 | 24 | 24 | 24 |

Notes: Simar-Wilson models estimated with 1,000 bootstrap replications. *p<0.1, **p<0.05, ***p<0.01. Standard errors in parentheses.

**Table A11. Further Simar-Wilson regression results of potential efficiency determinants, skilled birth attendance ratio poorest/richest.**

|  |  |  |  |  |  |  |  |  |  |  |  |  |  |  |  |  |  |
| --- | --- | --- | --- | --- | --- | --- | --- | --- | --- | --- | --- | --- | --- | --- | --- | --- | --- |
|  | (1) | (2) | (3) | (4) | (5) | (6) | (7) | (8) | (9) | (10) | (11) | (12) | (13) | (14) | (15) | (16) | (17) |
| *Organization of healthcare delivery and financing* |  |  |  |  |  |  |  |  |  |  |  |  |  |  |  |  |  |
| Out-of-pocket health expenditure (Perc.) | −0.017 |  |  |  |  |  |  |  |  |  |  |  |  |  |  |  | −0.005 |
|  | (0.086) |  |  |  |  |  |  |  |  |  |  |  |  |  |  |  | (0.006) |
| Hospital beds (per 1,000 people) |  | 0.264 |  |  |  |  |  |  |  |  |  |  |  |  |  |  | 0.09 |
|  |  | (0.472) |  |  |  |  |  |  |  |  |  |  |  |  |  |  | (0.073) |
| *Quality of governance* |  |  |  |  |  |  |  |  |  |  |  |  |  |  |  |  |  |
| Government effectiveness |  |  | 0.374 |  |  |  |  |  |  |  |  |  |  | 0.287 |  |  |  |
|  |  |  | (0.391) |  |  |  |  |  |  |  |  |  |  | (0.261) |  |  |  |
| Voice and accountability |  |  |  | 0.462* |  |  |  |  |  |  |  |  |  | 0.203 |  |  |  |
|  |  |  |  | (0.261) |  |  |  |  |  |  |  |  |  | (0.333) |  |  |  |
| Rule of law |  |  |  |  | 0.459 |  |  |  |  |  |  |  |  | 0.328 |  |  |  |
|  |  |  |  |  | (0.506) |  |  |  |  |  |  |  |  | (0.318) |  |  |  |
| Regulatory quality |  |  |  |  |  | 0.264 |  |  |  |  |  |  |  | −0.127 |  |  |  |
|  |  |  |  |  |  | (3.894) |  |  |  |  |  |  |  | (0.145) |  |  |  |
| Political stability and absence of violence/terrorism |  |  |  |  |  |  | 0.297 |  |  |  |  |  |  | 0.021 |  |  |  |
|  |  |  |  |  |  |  | (2.078) |  |  |  |  |  |  | (0.131) |  |  |  |
| Control of corruption |  |  |  |  |  |  |  | 0.472 |  |  |  |  |  | −0.399 |  |  |  |
|  |  |  |  |  |  |  |  | (1.712) |  |  |  |  |  | (0.318) |  |  |  |
| Average governance quality |  |  |  |  |  |  |  |  | 0.477 |  |  |  |  |  |  | 0.399** | 0.198 |
|  |  |  |  |  |  |  |  |  | (0.390) |  |  |  |  |  |  | (0.173) | (0.160) |
| *Quality of health system institutions* |  |  |  |  |  |  |  |  |  |  |  |  |  |  |  |  |  |
| Medium term sectoral vision in line with the government plan |  |  |  |  |  |  |  |  |  | 0.166 |  |  |  |  | 0.12 | −0.011 | −0.003 |
|  |  |  |  |  |  |  |  |  |  | (2.820) |  |  |  |  | (0.188) | (0.065) | (0.053) |
| Results-based management in the production of goods and services |  |  |  |  |  |  |  |  |  |  | 0.267 |  |  |  | 0.249 | 0.191* | 0.149* |
|  |  |  |  |  |  |  |  |  |  |  | (3.190) |  |  |  | (0.460) | (0.115) | (0.084) |
| Sectoral information systems |  |  |  |  |  |  |  |  |  |  |  | −0.132 |  |  | −0.250 | −0.151* | −0.110 |
|  |  |  |  |  |  |  |  |  |  |  |  | (3.454) |  |  | (0.381) | (0.090) | (0.074) |
| Average institutional quality |  |  |  |  |  |  |  |  |  |  |  |  | 0.239 |  |  |  |  |
|  |  |  |  |  |  |  |  |  |  |  |  |  | (3.751) |  |  |  |  |
| Constant | 1.578 | 0.547 | 1.064 | 0.873*** | 1.22 | 1.184 | 1.102 | 1.203 | 1.091** | 0.683 | 0.715 | 1.644 | 0.77 | 0.986*** | 0.591 | 0.948*** | 0.883** |
|  | (8.871) | (1.333) | (4.678) | (0.195) | (0.895) | (23.041) | (11.108) | (4.849) | (0.474) | (18.755) | (16.190) | (27.729) | (27.108) | (0.239) | (0.401) | (0.257) | (0.365) |
| Observations | 19 | 19 | 19 | 19 | 19 | 19 | 19 | 19 | 19 | 18 | 18 | 18 | 18 | 19 | 18 | 18 | 18 |

Notes: Simar-Wilson models estimated with 1,000 bootstrap replications. *p<0.1, **p<0.05, ***p<0.01. Standard errors in parentheses.

**Table A12. Further Simar-Wilson regression results of potential efficiency determinants, skilled birth attendance ratio rural/urban.**

|  |  |  |  |  |  |  |  |  |  |  |  |  |  |  |  |  |  |
| --- | --- | --- | --- | --- | --- | --- | --- | --- | --- | --- | --- | --- | --- | --- | --- | --- | --- |
|  | (1) | (2) | (3) | (4) | (5) | (6) | (7) | (8) | (9) | (10) | (11) | (12) | (13) | (14) | (15) | (16) | (17) |
| *Organization of healthcare delivery and financing* |  |  |  |  |  |  |  |  |  |  |  |  |  |  |  |  |  |
| Out-of-pocket health expenditure (Perc.) | −0.008 |  |  |  |  |  |  |  |  |  |  |  |  |  |  |  | −0.004 |
|  | (0.045) |  |  |  |  |  |  |  |  |  |  |  |  |  |  |  | (0.004) |
| Hospital beds (per 1,000 people) |  | 0.133 |  |  |  |  |  |  |  |  |  |  |  |  |  |  | 0.033 |
|  |  | (0.257) |  |  |  |  |  |  |  |  |  |  |  |  |  |  | (0.050) |
| *Quality of governance* |  |  |  |  |  |  |  |  |  |  |  |  |  |  |  |  |  |
| Government effectiveness |  |  | 0.181 |  |  |  |  |  |  |  |  |  |  | 0.081 |  |  |  |
|  |  |  | (1.171) |  |  |  |  |  |  |  |  |  |  | (0.128) |  |  |  |
| Voice and accountability |  |  |  | 0.09 |  |  |  |  |  |  |  |  |  | −0.221 |  |  |  |
|  |  |  |  | (1.680) |  |  |  |  |  |  |  |  |  | (0.154) |  |  |  |
| Rule of law |  |  |  |  | 0.232 |  |  |  |  |  |  |  |  | 0.372* |  |  |  |
|  |  |  |  |  | (0.883) |  |  |  |  |  |  |  |  | (0.201) |  |  |  |
| Regulatory quality |  |  |  |  |  | 0.026 |  |  |  |  |  |  |  | −0.074 |  |  |  |
|  |  |  |  |  |  | (2.637) |  |  |  |  |  |  |  | (0.084) |  |  |  |
| Political stability and absence of violence/terrorism |  |  |  |  |  |  | 0.166 |  |  |  |  |  |  | 0.113 |  |  |  |
|  |  |  |  |  |  |  | (0.716) |  |  |  |  |  |  | (0.078) |  |  |  |
| Control of corruption |  |  |  |  |  |  |  | 0.212 |  |  |  |  |  | −0.139 |  |  |  |
|  |  |  |  |  |  |  |  | (0.897) |  |  |  |  |  | (0.141) |  |  |  |
| Average governance quality |  |  |  |  |  |  |  |  | 0.231 |  |  |  |  |  |  | 0.199* | 0.080 |
|  |  |  |  |  |  |  |  |  | (0.259) |  |  |  |  |  |  | (0.115) | (0.107) |
| *Quality of health system institutions* |  |  |  |  |  |  |  |  |  |  |  |  |  |  |  |  |  |
| Medium term sectoral vision in line with the government plan |  |  |  |  |  |  |  |  |  | 0.036 |  |  |  |  | 0.024 | −0.025 | −0.011 |
|  |  |  |  |  |  |  |  |  |  | (0.764) |  |  |  |  | (0.318) | (0.047) | (0.037) |
| Results-based management in the production of goods and services |  |  |  |  |  |  |  |  |  |  | 0.059 |  |  |  | 0.084 | 0.076 | 0.053 |
|  |  |  |  |  |  |  |  |  |  |  | (1.597) |  |  |  | (0.371) | (0.071) | (0.060) |
| Sectoral information systems |  |  |  |  |  |  |  |  |  |  |  | −0.007 |  |  | −0.055 | −0.034 | −0.018 |
|  |  |  |  |  |  |  |  |  |  |  |  | (1.115) |  |  | (0.376) | (0.052) | (0.043) |
| Average institutional quality |  |  |  |  |  |  |  |  |  |  |  |  | 0.047 |  |  |  |  |
|  |  |  |  |  |  |  |  |  |  |  |  |  | (1.464) |  |  |  |  |
| Constant | 1.272 | 0.766*** | 1.068 | 1.073 | 1.113 | 1.114 | 1.023 | 1.07 | 1.069*** | 0.942 | 0.922 | 1.088 | 0.949 | 1.177*** | 0.879 | 0.992*** | 1.018*** |
|  | (3.517) | (0.211) | (5.621) | (10.412) | (2.243) | (15.025) | (1.942) | (2.710) | (0.399) | (10.048) | (9.914) | (9.783) | (10.287) | (0.142) | (0.831) | (0.186) | (0.212) |
| Observations | 21 | 21 | 21 | 21 | 21 | 21 | 21 | 21 | 21 | 19 | 19 | 19 | 19 | 21 | 19 | 19 | 19 |

Notes: Simar-Wilson models estimated with 1,000 bootstrap replications. *p<0.1, **p<0.05, ***p<0.01. Standard errors in parentheses.

**Table A13. Further Simar-Wilson regression results of potential efficiency determinants using total health expenditure per capita as input, life expectancy at birth.**

|  | (1) | (2) | (3) |
| --- | --- | --- | --- |
| Out-of-pocket health expenditure (Perc.) | 0.001 |  | 0.001 |
|  | (0.001) |  | (0.001) |
| Hospital beds (per 1,000 people) | 0.002 |  | −0.007 |
|  | (0.007) |  | (0.007) |
| Average governance quality |  | 0.003 | 0.020 |
|  |  | (0.014) | (0.017) |
| Average institutional quality |  | 0.010 | 0.007 |
|  |  | (0.012) | (0.011) |
| Constant | 0.922*** | 0.913*** | 0.907*** |
|  | (0.036) | (0.031) | (0.046) |
| Observations | 27 | 24 | 24 |
| Notes: Simar-Wilson models estimated with 1,000 bootstrap replications. *p<0.1, **p<0.05, ***p<0.01. Standard errors in parentheses. For specification (1), the very small coefficient and standard error for out-of-pocket health expenditure are rounded to three decimals. | | | |

**Table A14. Further Simar-Wilson regression results of potential efficiency determinants using total health expenditure per capita as input, life expectancy at age 60.**

|  | (1) | (2) | (3) |
| --- | --- | --- | --- |
| Out-of-pocket health expenditure (Perc.) | 0.001 |  | 0.001 |
|  | (0.002) |  | (0.002) |
| Hospital beds (per 1,000 people) | −0.009 |  | −0.016 |
|  | (0.015) |  | (0.019) |
| Average governance quality |  | −0.004 | 0.019 |
|  |  | (0.040) | (0.050) |
| Average institutional quality |  | 0.029 | 0.022 |
|  |  | (0.034) | (0.031) |
| Constant | 0.917*** | 0.841*** | 0.890*** |
|  | (0.086) | (0.087) | (0.122) |
| Observations | 27 | 24 | 24 |
| Notes: Simar-Wilson models estimated with 1,000 bootstrap replications. *p<0.1, **p<0.05, ***p<0.01. Standard errors in parentheses. For specifications (1) and (3), the very small coefficients for out-of-pocket health expenditure are rounded to three decimals. | | | |

**Table A15. Further Simar-Wilson regression results of potential efficiency determinants using total health expenditure per capita as input, under-five mortality.**

|  | (1) | (2) | (3) |
| --- | --- | --- | --- |
| Out-of-pocket health expenditure (Perc.) | −0.001 |  | 0.001 |
|  | (0.001) |  | (0.001) |
| Hospital beds (per 1,000 people) | 0.002 |  | −0.001 |
|  | (0.002) |  | (0.001) |
| Average governance quality |  | 0.004 | 0.005 |
|  |  | (0.003) | (0.003) |
| Average institutional quality |  | 0.001 | 0.001 |
|  |  | (0.002) | (0.002) |
| Constant | 0.988*** | 0.988*** | 0.991*** |
|  | (0.008) | (0.005) | (0.008) |
| Observations | 27 | 24 | 24 |
| Notes: Simar-Wilson models estimated with 1,000 bootstrap replications. *p<0.1, **p<0.05, ***p<0.01. Standard errors in parentheses. For specifications (1) and (3), the very small coefficients for out-of-pocket health expenditure and average institutional quality, and standard errors for out-of-pocket health expenditure, are rounded to three decimals. | | | |

**Table A16. Further Simar-Wilson regression results of potential efficiency determinants using total health expenditure per capita as input, DALYs lost.**

|  | (1) | (2) | (3) |
| --- | --- | --- | --- |
| Out-of-pocket health expenditure (Perc.) | 0.001 |  | 0.001 |
|  | (0.004) |  | (0.003) |
| Hospital beds (per 1,000 people) | 0.016 |  | −0.005 |
|  | (0.041) |  | (0.026) |
| Average governance quality |  | 0.041 | 0.061 |
|  |  | (0.070) | (0.060) |
| Average institutional quality |  | 0.054 | 0.051 |
|  |  | (0.040) | (0.040) |
| Constant | 0.870*** | 0.798** | 0.771*** |
|  | (0.302) | (0.312) | (0.154) |
| Observations | 27 | 24 | 24 |
| Notes: Simar-Wilson models estimated with 1,000 bootstrap replications. *p<0.1, **p<0.05, ***p<0.01. Standard errors in parentheses. | | | |

**Table A17. Further Simar-Wilson regression results of potential efficiency determinants using total health expenditure per capita as input, skilled birth attendance.**

|  | (1) | (2) | (3) |
| --- | --- | --- | --- |
| Out-of-pocket health expenditure (Perc.) | −0.020 |  | −0.017 |
|  | (0.018) |  | (0.014) |
| Hospital beds (per 1,000 people) | 0.544* |  | 0.241 |
|  | (0.302) |  | (0.176) |
| Average governance quality |  | 6.392*** | 0.010 |
|  |  | (1.428) | (0.205) |
| Average institutional quality |  | 0.671 | 0.144 |
|  |  | (0.580) | (0.125) |
| Constant | 1.550* | 8.935** | 1.299* |
|  | (0.917) | (3.666) | (0.759) |
| Observations | 26 | 23 | 23 |
| Notes: Simar and Wilson models estimated by 1000 bootstraps. *p<0.1, **p<0.05, ***p<0.01. Standard errors in parentheses | | | |

**Table A18. Further Simar-Wilson regression results of potential efficiency determinants using total health expenditure per capita as input, DPT immunization.**

|  | (1) | (2) | (3) |
| --- | --- | --- | --- |
| Out-of-pocket health expenditure (Perc.) | −0.002** |  | −0.001 |
|  | (0.001) |  | (0.001) |
| Hospital beds (per 1,000 people) | 0.005 |  | −0.001 |
|  | (0.009) |  | (0.009) |
| Average governance quality |  | 0.030* | 0.023 |
|  |  | (0.017) | (0.020) |
| Average institutional quality |  | 0.002 | 0.002 |
|  |  | (0.013) | (0.013) |
| Constant | 0.969*** | 0.910*** | 0.935*** |
|  | (0.045) | (0.035) | (0.055) |
| Observations | 27 | 24 | 24 |
| Notes: Simar-Wilson models estimated with 1,000 bootstrap replications. *p<0.1, **p<0.05, ***p<0.01. Standard errors in parentheses. For specification (3), the very small coefficient for hospital beds is rounded to three decimals. | | | |

**Table A19. Further Simar-Wilson regression results of potential efficiency determinants using total health expenditure per capita as input, skilled birth attendance ratio poorest/richest.**

|  | (1) | (2) | (3) |
| --- | --- | --- | --- |
| Out-of-pocket health expenditure (Perc.) | −0.007 |  | −0.003 |
|  | (0.007) |  | (0.006) |
| Hospital beds (per 1,000 people) | 0.159 |  | 0.114 |
|  | (0.135) |  | (0.082) |
| Average governance quality |  | 0.421** | 0.193 |
|  |  | (0.211) | (0.158) |
| Average institutional quality |  | 0.036 | 0.070 |
|  |  | (0.096) | (0.080) |
| Constant | 0.872*** | 0.951*** | 0.701** |
|  | (0.301) | (0.306) | (0.351) |
| Observations | 19 | 18 | 18 |
| Notes: Simar-Wilson models estimated with 1,000 bootstrap replications. *p<0.1, **p<0.05, ***p<0.01. Standard errors in parentheses. | | | |

**Table A20. Further Simar-Wilson regression results of potential efficiency determinants using total health expenditure per capita as input, skilled birth attendance ratio rural/urban.**

|  |  |  |  |
| --- | --- | --- | --- |
|  | (1) | (2) | (3) |
| Out-of-pocket health expenditure (Perc.) | −0.004 |  | −0.005 |
|  | (0.006) |  | (0.004) |
| Hospital beds (per 1,000 people) | 0.083 |  | 0.033 |
|  | (0.142) |  | (0.058) |
| Average governance quality |  | 0.197 | 0.062 |
|  |  | (0.709) | (0.120) |
| Average institutional quality |  | 0.001 | 0.016 |
|  |  | (0.183) | (0.046) |
| Constant | 0.966*** | 1.019 | 1.039*** |
|  | (0.357) | (1.573) | (0.264) |
| Observations | 21 | 19 | 19 |
| Notes: Simar-Wilson models estimated with 1,000 bootstrap replications. *p<0.1, **p<0.05, ***p<0.01. Standard errors in parentheses. | | | |

**Table A21. Further Simar-Wilson regression results of potential efficiency determinants using pooled health expenditure per capita as input, life expectancy at birth.**

|  | (1) | (2) | (3) |
| --- | --- | --- | --- |
| Out-of-pocket health expenditure (Perc.) | 0.001 |  | 0.001 |
|  | (0.001) |  | (0.001) |
| Hospital beds (per 1,000 people) | 0.002 |  | −0.007 |
|  | (0.007) |  | (0.007) |
| Average governance quality |  | 0.003 | 0.022 |
|  |  | (0.013) | (0.016) |
| Average institutional quality |  | 0.013 | 0.010 |
|  |  | (0.012) | (0.011) |
| Constant | 0.909*** | 0.905*** | 0.887*** |
|  | (0.035) | (0.030) | (0.044) |
| Observations | 27 | 24 | 24 |
| Notes: Simar-Wilson models estimated with 1,000 bootstrap replications. *p<0.1, **p<0.05, ***p<0.01. Standard errors in parentheses. | | | |

**Table A22. Further Simar-Wilson regression results of potential efficiency determinants using pooled health expenditure per capita as input, life expectancy at age 60.**

|  | (1) | (2) | (3) |
| --- | --- | --- | --- |
| Out-of-pocket health expenditure (Perc.) | 0.001 |  | 0.001 |
|  | (0.002) |  | (0.002) |
| Hospital beds (per 1,000 people) | −0.008 |  | −0.017 |
|  | (0.018) |  | (0.023) |
| Average governance quality |  | −0.010 | 0.023 |
|  |  | (0.045) | (0.053) |
| Average institutional quality |  | 0.033 | 0.025 |
|  |  | (0.040) | (0.036) |
| Constant | 0.897*** | 0.848*** | 0.868*** |
|  | (0.101) | (0.097) | (0.143) |
| Observations | 27 | 24 | 24 |
| Notes: Simar-Wilson models estimated with 1,000 bootstrap replications. *p<0.1, **p<0.05, ***p<0.01. Standard errors in parentheses. | | | |

**Table A23. Further Simar-Wilson regression results of potential efficiency determinants using pooled health expenditure per capita as input, under-five mortality.**

|  | (1) | (2) | (3) |
| --- | --- | --- | --- |
| Out-of-pocket health expenditure (Perc.) | -0.001 |  | 0.001 |
|  | (0.001) |  | (0.001) |
| Hospital beds (per 1,000 people) | 0.002 |  | −0.001 |
|  | (0.002) |  | (0.001) |
| Average governance quality |  | 0.004* | 0.006** |
|  |  | (0.003) | (0.003) |
| Average institutional quality |  | 0.001 | 0.001 |
|  |  | (0.002) | (0.002) |
| Constant | 0.987*** | 0.986*** | 0.988*** |
|  | (0.007) | (0.005) | (0.008) |
| Observations | 27 | 24 | 24 |
| Notes: Simar-Wilson models estimated with 1,000 bootstrap replications. *p<0.1, **p<0.05, ***p<0.01. Standard errors in parentheses. For specifications (1) and (3), the very small coefficient and standard error for out-of-pocket health expenditure are rounded to three decimals. | | | |

**Table A24. Further Simar-Wilson regression results of potential efficiency determinants using pooled health expenditure per capita as input, DALYs lost.**

|  | (1) | (2) | (3) |
| --- | --- | --- | --- |
| Out-of-pocket health expenditure (Perc.) | 0.001 |  | 0.002 |
|  | (0.003) |  | -0.003 |
| Hospital beds (per 1,000 people) | 0.017 |  | −0.005 |
|  | (0.032) |  | -0.024 |
| Average governance quality |  | 0.043 | 0.067 |
|  |  | (0.050) | (0.060) |
| Average institutional quality |  | 0.064 | 0.060 |
|  |  | (0.040) | (0.040) |
| Constant | 0.857*** | 0.771*** | 0.730*** |
|  | (0.169) | (0.098) | (0.146) |
| Observations | 27 | 24 | 24 |
| Notes: Simar-Wilson models estimated with 1,000 bootstrap replications. *p<0.1, **p<0.05, ***p<0.01. Standard errors in parentheses. | | | |

**Table A25. Further Simar-Wilson regression results of potential efficiency determinants using pooled health expenditure per capita as input, skilled birth attendance.**

|  | (1) | (2) | (3) |
| --- | --- | --- | --- |
|  | β/SE | β/SE | β/SE |
| Out-of-pocket health expenditure (Perc.) | −0.015 |  | −0.007 |
|  | (0.014) |  | (0.005) |
| Hospital beds (per 1,000 people) | 0.509 |  | 0.134 |
|  | (0.323) |  | (0.090) |
| Average governance quality |  | 0.381 | 0.09 |
|  |  | (0.297) | (0.119) |
| Average institutional quality |  | 0.123 | 0.144* |
|  |  | (0.122) | (0.078) |
| Constant | 1.317* | 1.007*** | 0.861*** |
|  | (0.702) | (0.358) | (0.289) |
| Observations | 26 | 23 | 23 |
| Notes: Simar and Wilson models estimated by 1000 bootstraps. *p<0.1, **p<0.05, ***p<0.01. Standard errors in parentheses | | | |

**Table A26. Further Simar-Wilson regression results of potential efficiency determinants using pooled health expenditure per capita as input, DPT immunization.**

|  | (1) | (2) | (3) |
| --- | --- | --- | --- |
| Out-of-pocket health expenditure (Perc.) | −0.002** |  | −0.001 |
|  | (0.001) |  | (0.001) |
| Hospital beds (per 1,000 people) | 0.006 |  | −0.001 |
|  | (0.009) |  | (0.009) |
| Average governance quality |  | 0.038** | 0.032 |
|  |  | (0.016) | (0.020) |
| Average institutional quality |  | 0.012 | 0.012 |
|  |  | (0.012) | (0.013) |
| Constant | 0.966*** | 0.881*** | 0.903*** |
|  | (0.047) | (0.032) | (0.054) |
| Observations | 27 | 24 | 24 |
| Notes: Simar-Wilson models estimated with 1,000 bootstrap replications. *p<0.1, **p<0.05, ***p<0.01. Standard errors in parentheses. For specification (3), the very small coefficient for hospital beds is rounded to three decimals. | | | |

**Table A27. Further Simar-Wilson regression results of potential efficiency determinants using pooled health expenditure per capita as input, skilled birth attendance ratio poorest/richest.**

|  | (1) | (2) | (3) |
| --- | --- | --- | --- |
| Out-of-pocket health expenditure (Perc.) | −0.007 |  | −0.002 |
|  | (0.007) |  | (0.005) |
| Hospital beds (per 1,000 people) | 0.166 |  | 0.110 |
|  | (0.136) |  | (0.076) |
| Average governance quality |  | 0.425** | 0.221 |
|  |  | (0.204) | (0.145) |
| Average institutional quality |  | 0.055 | 0.088 |
|  |  | (0.087) | (0.074) |
| Constant | 0.883*** | 0.884*** | 0.626* |
|  | (0.335) | (0.276) | (0.344) |
| Observations | 19 | 18 | 18 |
| Notes: Simar-Wilson models estimated with 1,000 bootstrap replications. *p<0.1, **p<0.05, ***p<0.01. Standard errors in parentheses. | | | |

**Table A28. Further Simar-Wilson regression results of potential efficiency determinants using pooled health expenditure per capita as input, skilled birth attendance ratio rural/urban.**

|  | (1) | (2) | (3) |
| --- | --- | --- | --- |
| Out-of-pocket health expenditure (Perc.) | −0.004 |  | −0.003 |
|  | (0.004) |  | (0.003) |
| Hospital beds (per 1,000 people) | 0.077 |  | 0.022 |
|  | (0.067) |  | (0.043) |
| Average governance quality |  | 0.181* | 0.094 |
|  |  | (0.097) | (0.090) |
| Average institutional quality |  | 0.024 | 0.034 |
|  |  | (0.042) | (0.038) |
| Constant | 0.948*** | 0.911*** | 0.935*** |
|  | (0.182) | (0.142) | (0.190) |
| Observations | 21 | 19 | 19 |
| Notes: Simar-Wilson models estimated with 1,000 bootstrap replications. *p<0.1, **p<0.05, ***p<0.01. Standard errors in parentheses. | | | |

**Table A29. Summary results of conditional order-m regression estimations.**

| Output and environmental variables | P-value | Interpretation: Higher efficiency is associated with… |
| --- | --- | --- |
| Life expectancy at birth |  |  |
| Out-of-pocket health expenditure (Perc.) | 0.008*** | Lower share |
| Hospital beds (per 1,000 people) | 0.002*** | More beds |
| Average governance quality | 0.001*** | Higher governance quality |
| Average institutional quality | 0.007*** | Higher institutional quality |
|  |  |  |
| Life expectancy at age 60 |  |  |
| Out-of-pocket health expenditure (Perc.) | 0.0001*** | Lower share |
| Hospital beds (per 1,000 people) | 0.0001*** | More beds |
| Average governance quality | 0.086* | Higher governance quality |
| Average institutional quality | 1.000 | *No effect* |
|  |  |  |
| Under-five mortaility (per 1,000) |  |  |
| Out-of-pocket health expenditure (Perc.) | 0.0001*** | Lower share |
| Hospital beds (per 1,000 people) | 0.075* | Relationship varies according to level of environmental variable |
| Average governance quality | 0.0001*** | Higher governance quality |
| Average institutional quality | 0.0001*** | Higher institutional quality |
|  |  |  |
| DALYs lost (per 100,000) |  |  |
| Out-of-pocket health expenditure (Perc.) | 0.027** | Lower share |
| Hospital beds (per 1,000 people) | 0.007*** | More beds |
| Average governance quality | 0.001*** | Higher governance quality |
| Average institutional quality | 0.0001*** | Relationship varies according to level of environmental variable |
|  |  |  |
| Skilled birth attendance |  |  |
| Out-of-pocket health expenditure (Perc.) | 0.570 | *No effect* |
| Hospital beds (per 1,000 people) | 0.067* | Relationship varies according to level of environmental variable |
| Average governance quality | 0.010** | Higher governance quality |
| Average institutional quality | 0.511 | *No effect* |
|  |  |  |
| DPT immunization |  |  |
| Out-of-pocket health expenditure (Perc.) | 0.606 | *No effect* |
| Hospital beds (per 1,000 people) | 0.083* | More beds |
| Average governance quality | 0.057* | Higher governance quality |
| Average institutional quality | 0.019** | Higher institutional quality |
|  |  |  |
| Skilled birth attendance ratio poorest/richest |  |  |
| Out-of-pocket health expenditure (Perc.) | 1.000 | *No effect* |
| Hospital beds (per 1,000 people) | 0.0001*** | More beds |
| Average governance quality | 0.0001*** | Higher governance quality |
| Average institutional quality | 0.804 | *No effect* |
|  |  |  |
| Skilled birth attendance ratio rural/urban |  |  |
| Out-of-pocket health expenditure (Perc.) | 0.020** | Relationship varies according to level of environmental variable |
| Hospital beds (per 1,000 people) | 0.121 | *No effect* |
| Average governance quality | 0.004*** | Higher governance quality |
| Average institutional quality | 0.025** | Higher institutional quality |

Notes: Conditional order-m regression models (p-values). *p<0.1, **p<0.05, ***p<0.01. The dependent variable in the model for each output corresponds to the ratio of order-m conditional (on environmental variables) to unconditional estimates. The p-values for the tests of statistical significance of environmental variables are obtained from nonparametric regressions estimated with 1,000 bootstrap replications.

**Figure A1. Partial regression plot of conditional order-m regression of efficiency ratio on potential efficiency determinants, life expectancy at birth.**


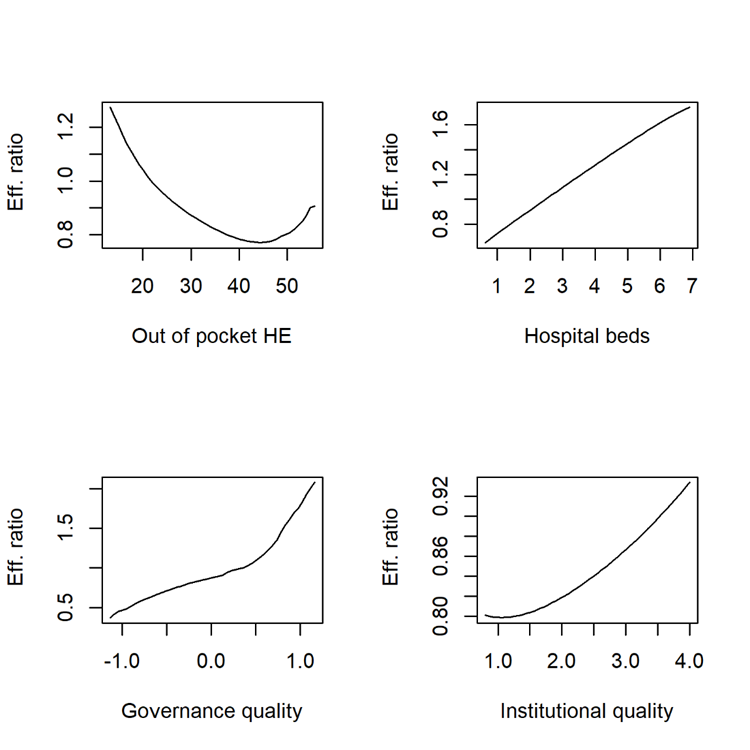


Notes: Eff. ratio refers to the ratio of conditional efficiency estimates to unconditional efficiency estimates. See also Table A29. For the definition of potential determinant (environmental) variables, see main text.

**Figure A2. Partial regression plot of conditional order-m regression of efficiency ratio on potential efficiency determinants, life expectancy at age 60.**


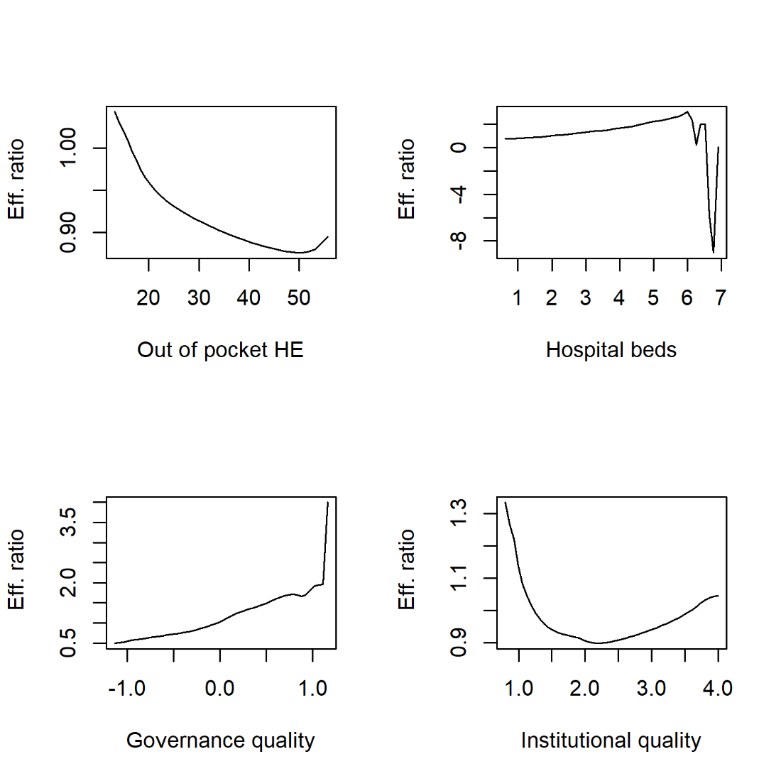


Notes: Eff. ratio refers to the ratio of conditional efficiency estimates to unconditional efficiency estimates. See also Table A29. For the definition of potential determinant (environmental) variables, see main text.

**Figure A3. Partial regression plot of conditional order-m regression of efficiency ratio on potential efficiency determinants, under-five mortality.**


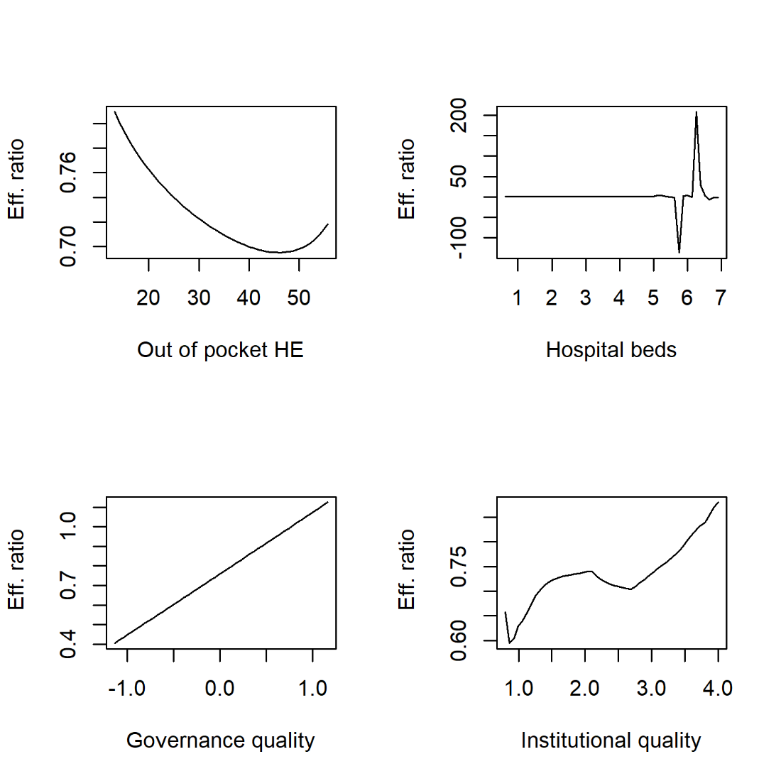


Notes: Eff. ratio refers to the ratio of conditional efficiency estimates to unconditional efficiency estimates. See also Table A29. For the definition of potential determinant (environmental) variables, see main text.

**Figure A4. Partial regression plot of conditional order-m regression of efficiency ratio on potential efficiency determinants, DALYs lost.**


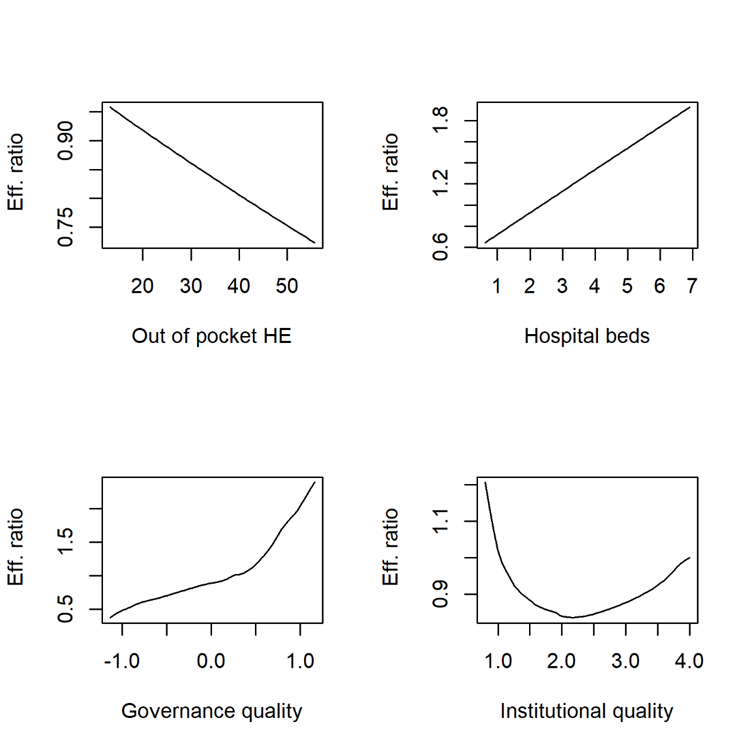


Notes: Eff. ratio refers to the ratio of conditional efficiency estimates to unconditional efficiency estimates. See also Table A29. For the definition of potential determinant (environmental) variables, see main text.

**Figure A5. Partial regression plot of conditional order-m regression of efficiency ratio on potential efficiency determinants, skilled birth attendance.**


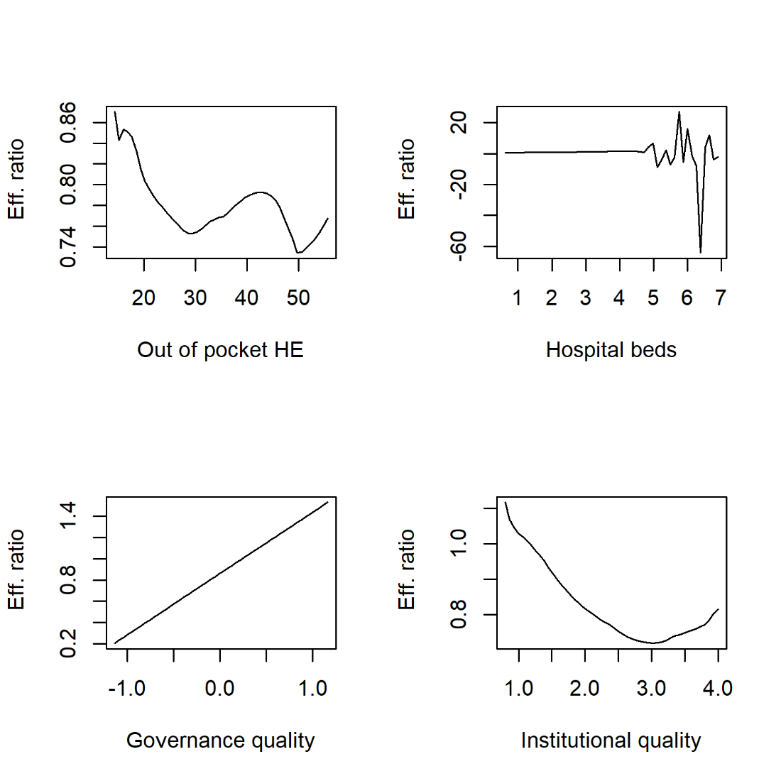


Notes: Eff. ratio refers to the ratio of conditional efficiency estimates to unconditional efficiency estimates. See also Table A29. For the definition of potential determinant (environmental) variables, see main text.

**Figure A6. Partial regression plot of conditional order-m regression of efficiency ratio on potential efficiency determinants, DPT immunization.**


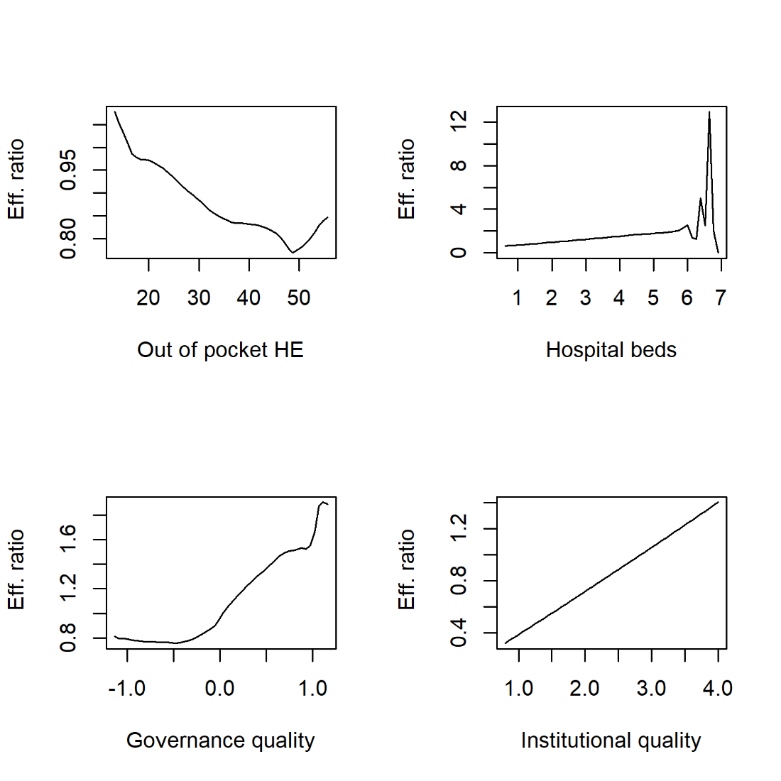


Notes: Eff. ratio refers to the ratio of conditional efficiency estimates to unconditional efficiency estimates. See also Table A29. For the definition of potential determinant (environmental) variables, see main text.

**Figure A7. Partial regression plot of conditional order-m regression of efficiency ratio on potential efficiency determinants, skilled birth attendance ratio poorest/richest.**


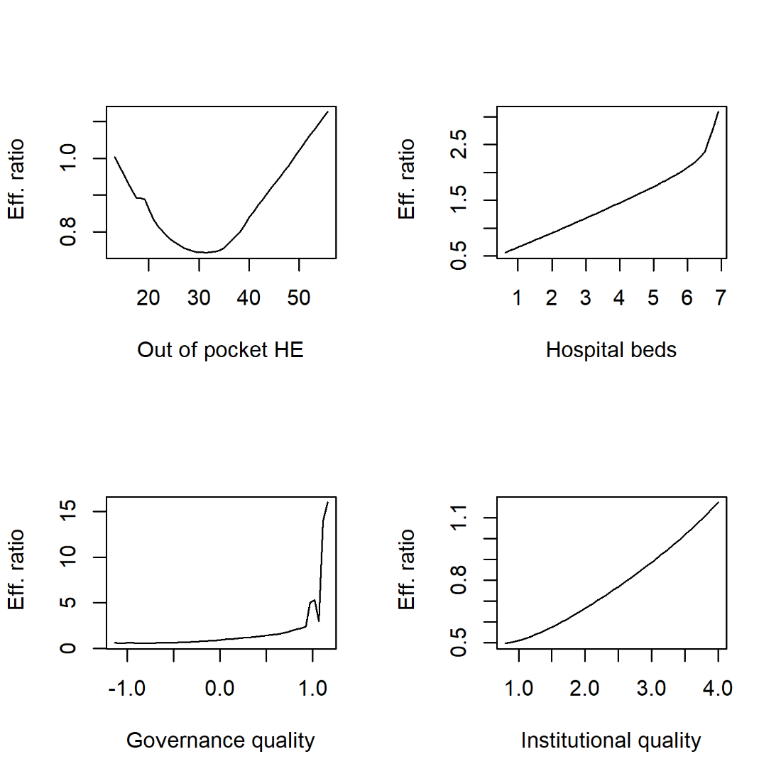


Notes: Eff. ratio refers to the ratio of conditional efficiency estimates to unconditional efficiency estimates. See also Table A29. For the definition of potential determinant (environmental) variables, see main text.

**Figure A8. Partial regression plot of conditional order-m regression of efficiency ratio on potential efficiency determinants, skilled birth attendance ratio rural/urban.**


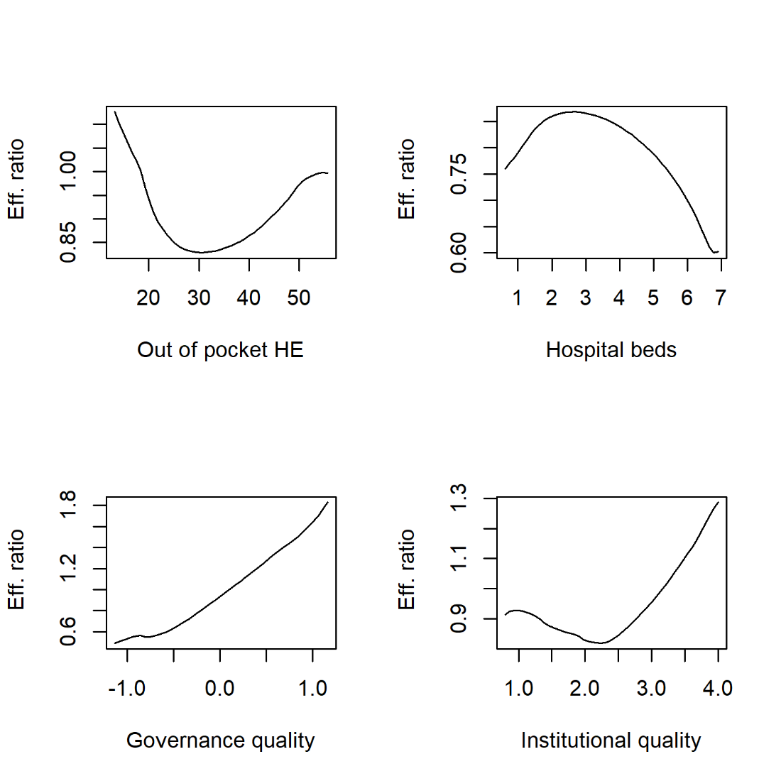


Notes: Eff. ratio refers to the ratio of conditional efficiency estimates to unconditional efficiency estimates. See also Table A29. For the definition of potential determinant (environmental) variables, see main text.
